# Depressive and Negative Symptoms in the Early and Established Stages of Schizophrenia: Integrating Structural Brain Alterations, Cognitive Performance, and Plasma Interleukin 6 Levels

**DOI:** 10.1016/j.bpsgos.2024.100429

**Published:** 2024-12-02

**Authors:** Fabiana Corsi-Zuelli, Gary Donohoe, Siân Lowri Griffiths, Cristina M. Del-Ben, Andrew J. Watson, Tom Burke, Paris A. Lalousis, Declan McKernan, Derek Morris, John Kelly, Colm McDonald, Saahithh R. Patlola, Carmine Pariante, Nicholas M. Barnes, Golam M. Khandaker, John Suckling, Bill Deakin, Rachel Upthegrove, Maria R. Dauvermann

**Affiliations:** a Institute for Mental Health, School of Psychology, University of Birmingham, Birmingham, United Kingdom; b Department of Neurosciences and Behaviour, Ribeirão Preto Medical School, University of São Paulo, São Paulo, Brazil; c Centre for Neuroimaging, Cognition and Genomics, School of Psychology, University of Galway, Galway, Ireland; d Department of Clinical and Motor Neuroscience, University College London, Queen Square Institute of Neurology, London, United Kingdom; e Department of Psychosis Studies, Institute of Psychiatry, Psychology, and Neuroscience, King’s College London, London, United Kingdom; f Pharmacology & Therapeutics, School of Medicine, University of Galway, Galway, Ireland; g Department of Psychological Medicine, Institute of Psychiatry, Psychology, and Neuroscience, King’s College London, London, United Kingdom; h College of Medical and Dental Sciences, University of Birmingham, Birmingham, United Kingdom; i MRC Integrative Epidemiology Unit, Population Health Sciences, Bristol Medical School, University of Bristol, Bristol, United Kingdom; j Centre for Academic Mental Health, Population Health Sciences, Bristol Medical School, University of Bristol, Bristol, United Kingdom; k NIHR Bristol Biomedical Research Centre, University Hospitals Bristol and Weston NHS Foundation Trust, Bristol, United Kingdom; l Department of Psychiatry, University of Cambridge, Cambridge, United Kingdom; m Cambridgeshire and Peterborough NHS Foundation Trust, Cambridge, United Kingdom; n Division of Neuroscience and Experimental Psychology, Faculty of Biology, Medicine and Health, School of the Biological Sciences, University of Manchester, Manchester, United Kingdom; o Department of Psychiatry, University of Oxford, Oxford, United Kingdom

**Keywords:** Depressive and negative symptoms, General cognitive function, Plasma interleukin 6, Schizophrenia, Structural equation modeling, Structural MRI

## Abstract

**Background:**

Depressive and negative symptoms are related to poor functional outcomes in schizophrenia. Cognitive deficits, reduced brain cortical thickness and volumes, and inflammation may contribute to depressive and negative symptoms, but pharmacological treatment and disease progression may confound the associations.

**Methods:**

We evaluated whether higher plasma interleukin 6 (IL-6) levels would be associated with more severe negative or depressive symptoms in schizophrenia and explored illness stage utilizing early (BeneMin [Benefit of Minocycline on Negative Symptoms of Psychosis: Extent and Mechanism], *n* = 201, 72.8% male) and established (iRELATE [Immune Response & Social Cognition in Schizophrenia], *n* = 94, 67.3% male) schizophrenia cohorts. Using structural equation modeling in a subsample (iRELATE: *n* = 42, 69.0% male; BeneMin: *n* = 102, 76.5% male) with data on structural brain metrics (cortical thickness and volume), general cognitive performance, and plasma IL-6 levels, we assessed the interrelationships between these variables on depressive and negative symptom severity in early and established schizophrenia samples combined and in early schizophrenia only. All analyses were adjusted for sex, age, and chlorpromazine equivalent dose.

**Results:**

Higher plasma IL-6 levels were related to more severe depressive symptoms in early schizophrenia (*p* < .05) and negative symptoms in established schizophrenia (*p* < .05). Structural equation modeling findings in early and established schizophrenia samples combined and early schizophrenia only showed that the interrelationship between higher plasma IL-6 levels, structural brain metrics, and general cognitive performance did not predict the severity of depressive and negative symptoms (*p* > .05). Higher plasma IL-6 levels and lower general cognitive performance were associated with reduced brain metrics (*p* < .05).

**Conclusions:**

Our results indicate that higher plasma IL-6 levels may be differently associated with the severity of depressive and negative symptoms dependent on the illness stage. Future work identifying elevated levels of inflammation in larger samples may allow stratification and personalized intervention by subgroups who are at risk of poor outcomes.

## Plain Language Summary

In this study, we examined whether elevated levels of plasma IL-6, an inflammatory protein, are associated with more severe depressive and negative symptoms in schizophrenia. Using data from Early-stage (BeneMin) and Established (iRELATE) cohorts, we found that higher plasma IL-6 levels were associated with more severe depressive symptoms in Early schizophrenia and more severe negative symptoms in Established schizophrenia. However, the interrelationship between plasma IL-6, brain structure, and cognitive performance did not predict symptom severity. These findings suggest that high plasma IL-6 levels may affect symptoms differently depending on the stage of schizophrenia, supporting the need for targeted interventions based on symptoms and inflammation profiles.

Schizophrenia spectrum disorders are associated with a wide range of symptoms, such as positive (hallucinations and delusions) and negative (blunted affect, avolition, alogia, anhedonia, associability) symptoms as well as cognitive deficits (e.g., learning, memory, and executive function) (1). In addition, depressive symptoms are common during the early stages of psychosis (2), affecting approximately 40% of individuals (3). It is widely established that negative, depressive, and cognitive symptoms in individuals with schizophrenia contribute to poor functional outcomes, including significantly increased rates of hospitalization, treatment resistance, relapse, and suicide (2,4, 5, 6, 7, 8). Identifying and differentiating negative from depressive symptoms may have clinical implications for prognosis, reducing and preventing poor long-term outcomes (8,9).

Alterations in brain structure may underlie the occurrence of negative and depressive symptoms in individuals with schizophrenia and major depressive disorder (MDD) (10, 11, 12, 13, 14, 15, 16, 17, 18, 19, 20, 21). For example, findings from the ENIGMA (Enhancing Neuro Imaging Genetics through Meta Analysis) Consortium suggest that adults with schizophrenia and MDD may share a common profile of reduced cortical thickness (CT) in brain regions (such as the caudal and rostral portions of the anterior cingulate and the middle frontal, lateral and medial orbitofrontal, and middle temporal) compared with healthy control individuals (10,22, 23, 24, 25), with larger effect sizes in schizophrenia than MDD (24). These regions have been implicated in deficits in emotion regulation, social cognition, sensory integration, and reward-related motivation and anticipation (10,22, 23, 24, 25).

Immune system dysregulation is one potential mechanism that may explain relationships between altered brain structure and negative, depressive, and cognitive symptoms in individuals with schizophrenia. The neuroinflammatory hypothesis proposes that elevated levels of peripheral cytokines such as interleukin 6 (IL-6) cross the blood-brain barrier and induce changes in brain structure and function that may contribute to the generation of negative, depressive, and cognitive symptoms in individuals with schizophrenia and MDD (26,27). Support for immune dysregulation has been provided by meta-analyses (28,29) that have consistently shown that peripheral IL-6 levels are mildly increased in individuals with schizophrenia at the early and established stages as well as in individuals with MDD compared with healthy control participants. Specifically, higher peripheral IL-6 levels prior to the onset of the first episode of psychosis or depression (30,31) and before treatment initiation (28,29,32,33) suggest that increased peripheral IL-6 levels may be associated with the onset of psychotic and depressive symptoms. Furthermore, findings from Mendelian randomization (34, 35, 36) studies suggest that genetically determined elevated peripheral IL-6 levels may causally contribute to the development of both schizophrenia and MDD, with therapeutic potential shown in recent trials (37,38). Elevated levels of peripheral IL-6 may also be related to general cognitive deficits in individuals with schizophrenia (39) and MDD (39,40). However, it is not well known how these factors may be interrelated. Therefore, we focused our study on peripheral IL-6 levels.

There is some evidence that elevated peripheral IL-6 levels are related to alterations in brain structure in individuals with schizophrenia (41). For example, one recent study found that gray matter volume loss in temporal, hippocampal, and anterior cingulate areas in individuals with established schizophrenia was particularly evident in those who had increased peripheral IL-6 levels (42). In another study of individuals with established schizophrenia, reduced CT of the bilateral Broca’s area and temporal gyrus was related to higher peripheral IL-6 levels (43). However, in individuals with first-episode psychosis, whole-brain gray matter volume or reduced CT in the bilateral middle frontal gyrus was not associated with elevated peripheral IL-6 levels (44). Part of the inconsistency may be explained by disease heterogeneity and confounding factors, such as antipsychotic medication (44), which most studies do not adjust for. To increase our insight into the mechanistic processes that contribute to negative and depressive symptoms, the interrelationships between these factors need to be assessed together.

We evaluated whether higher plasma IL-6 levels would be associated with more severe negative or depressive symptoms in schizophrenia and explored how these associations might differ across stages of illness by utilizing early and established schizophrenia cohorts. To gain a better understanding of the potential underlying neurobiological and neurocognitive mechanisms, we examined the relationships between plasma IL-6 levels, general cognitive performance, and structural brain metrics (CT and volume) and depressive and negative symptom severity.

We predicted that elevated plasma IL-6 levels would be related to greater depressive and negative symptom severity, particularly in individuals with early-stage schizophrenia, when the influence of antipsychotic medication and other illness-related confounders is less pronounced. In addition, we expected that the relationship between elevated plasma IL-6 levels and greater depressive and negative symptom severity in individuals with early schizophrenia would occur through their association with reduced structural brain metrics of CT and volume as well as lower general cognitive performance.

## Methods and Materials

### Participants

#### BeneMin Study

We included baseline data from individuals with an acute episode of psychosis within 5 years of onset of schizophrenia spectrum as part of the BeneMin (Benefit of Minocycline on Negative Symptoms of Psychosis: Extent and Mechanism) clinical trial (45), hereafter called early schizophrenia.

BeneMin was a double-blind, randomized controlled trial testing the potential benefit of the anti-inflammatory minocycline on negative symptoms and cognition in individuals who were experiencing an acute episode of psychosis (schizophrenia, schizophreniform, or schizoaffective psychosis). Two hundred seven participants ages 16 to 35 years (46) were recruited. Participants were on stable antipsychotic medication. See Supplement section 2.1.1 for further details.

#### iRELATE Study

We included data from individuals with established schizophrenia from the iRELATE (Immune Response & Social Cognition in Schizophrenia) study. One hundred four individuals aged between 18 and 65 years were recruited (47, 48, 49). Individuals with schizophrenia from the iRELATE study were classified as chronic schizophrenia if the duration of illness was 12 months or more and the patient was clinically stable at the time of assessment, hereafter called established schizophrenia.

iRELATE aimed to investigate the impact of environment, genes, and the immune system on brain structure and function in schizophrenia. All participants were required to be clinically stable at the time of assessment. See Supplement section 2.1.2 for further details.

### Study Procedure

**Figure 1:**
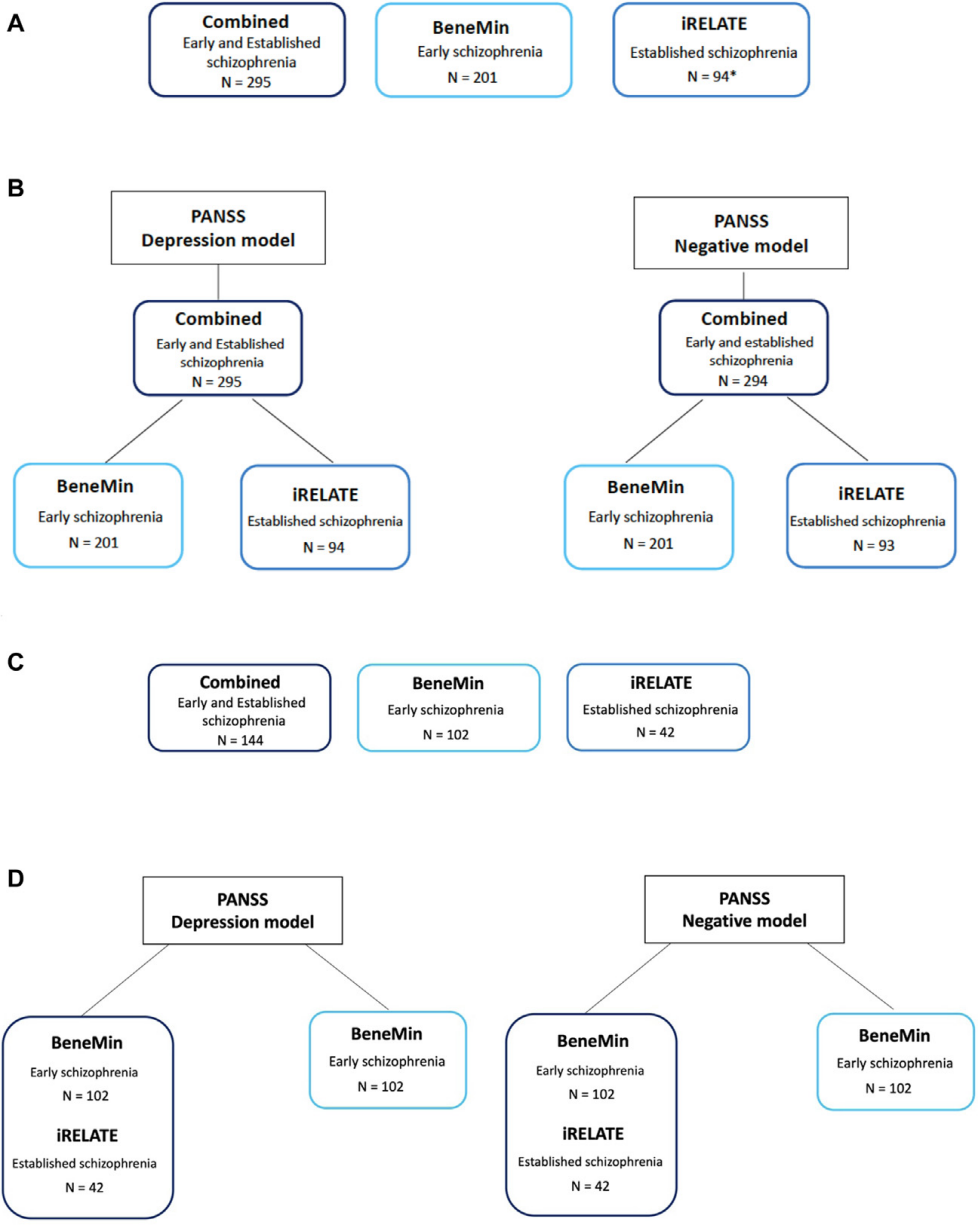
Sample size for individuals with early schizophrenia (BeneMin [Benefit of Minocycline on Negative Symptoms of Psychosis: Extent and Mechanism] study) and established schizophrenia (iRELATE [Immune Response & Social Cognition in Schizophrenia] study) and analytic models for general linear models (GLMs) and structural equation modeling (SEM). **(A)** Sample size for GLM. The sample size for GLM analyses comprised individuals with early (BeneMin sample, *n* = 201) and established (iRELATE sample, *n* = 94∗) schizophrenia who had complete data for the outcome measures (depressive or negative symptoms) and the independent variable, plasma interleukin 6 (IL-6) levels. ^∗^In the Positive and Negative Syndrome Scale (PANSS) depression model, data from 94 individuals with established schizophrenia were available, whereas for the PANSS negative model, data from 1 individual was missing. **(B)** Two models for GLM analyses. For the GLM analyses, we tested associations between plasma IL-6 and our main outcomes (PANSS depression and PANSS negative) in samples of individuals with early (BeneMin sample, *n* = 201) and established (iRELATE sample, *n* = 94∗) schizophrenia combined and separately. For all analyses across the 3 groupings, plasma IL-6 levels were natural log-transformed and *z*-scored. Thus, regression coefficients and 95% CIs represent changes per standard deviation of the exposure. Both unadjusted and adjusted (sex, age, chlorpromazine equivalent dose) models were performed. Results were considered significant at *p* < .05. **(C)** Sample size for SEM analyses. For the SEM analyses, only individuals with complete data on plasma IL-6 levels, CAT12-derived structural brain metrics of cortical thickness and volume, general cognitive performance, and severity of PANSS depressive or negative symptoms were included (early schizophrenia from BeneMin, *n* = 102; established schizophrenia from iRELATE, *n* = 42), totaling 144 individuals. **(D)** Two integrative models for SEM analyses. Our primary outcome measures in SEM analyses were the severity of depressive symptoms (PANSS depression model) and negative symptoms (PANSS negative model). Path analyses with composite scores were performed for both models (PANSS depression and PANSS negative) in separate groupings: 1) individuals with early schizophrenia (BeneMin study) combined with individuals with established schizophrenia (iRelate study), and 2) individuals with early schizophrenia only (BeneMin study). We were precluded from performing path analyses with composite scores in individuals with established schizophrenia only due to the limited sample size. Both unadjusted and adjusted (sex, age, chlorpromazine equivalent dose) models were performed.

Plasma IL-6 levels and the outcome variables of depressive and negative symptom severity (measured by the Positive and Negative Syndrome Scale [PANSS]) were available for 201 individuals with early schizophrenia and 94 individuals with established schizophrenia. This sample (combined and separately) was used to evaluate the associations between plasma IL-6 levels and depressive and negative symptoms using general linear models (GLMs) (Figure 1A, B).

Complete data for plasma IL-6 levels, cognitive and brain imaging variables, and depressive and negative symptoms were available for 102 individuals with early schizophrenia and 42 individuals with established schizophrenia. This sample (combined and early schizophrenia only) was used to examine the relationships between plasma IL-6 levels, general cognitive performance, and structural brain metrics (CT and volume) and depressive and negative symptoms using structural equation modeling (SEM) (Figure 1C, D).

#### Clinical Assessment

The severity of positive symptoms, negative symptoms, and general psychopathology of schizophrenia were assessed using the PANSS (50) (see Supplement section 2.2.1 for further details).

#### Clinical Assessment of Depressive Symptoms

Depressive symptoms were measured using 4 items of the PANSS general scale (PANSS-G1: somatic concern, PANSS-G2: anxiety, PANSS-G3: guilty feelings, and PANSS-G6: depression). The Calgary Depression Scale for Schizophrenia (51) was available for the BeneMin study, while the Hamilton Rating Scale for Depression (52) was available for the iRELATE study.

Previous research has demonstrated the validity of the PANSS depression scale (53, 54, 55), showing a strong relationship with other depression assessment tools, such as the Hamilton Rating Scale for Depression (*r* = 0.62) and Calgary Depression Scale for Schizophrenia (*r* = 0.66) (54). Congruently, PANSS-depression scores were strongly correlated with Hamilton Rating Scale for Depression scores in the iRELATE study (rho = 0.60, *p* < .001, *n* = 103) and with Calgary Depression Scale for Schizophrenia scores in the BeneMin study (rho = 0.66, *p* < .001, *n* = 205).

#### General Cognitive Performance

General cognitive performance in both cohorts was assessed by estimating IQ using the Wechsler Adult Intelligence Scale, third edition, comprising Digit Symbol, Information, Block Design, and Arithmetic (56). For further details, see Supplement section 2.2.2.

#### Magnetic Resonance Imaging: Acquisition and Image Processing

Structural magnetic resonance imaging (MRI) data for individuals with early and established schizophrenia were obtained using 3T MRI scanners. Detailed MRI acquisition and preprocessing steps are presented in Supplement section 2.2.3.1.

#### MRI: Structural MRI Analysis

Scans were processed using the open-source CAT-12 toolbox (https://neuro-jena.github.io/cat12-help/) for SPM12 software (http://www.fil.ion.ucl.ac.uk/spm/) within MATLAB (The MathWorks, Inc.). See Supplement section 2.2.3.2 for further information.

#### MRI: Region of Interest Selection

We selected 8 a priori regions of interest (ROIs) for CT and volume based on their association with either schizophrenia, depressive or negative symptoms, general cognitive performance, or inflammation (Table S1). Seven ROIs were chosen for CT, namely the caudal and rostral anterior cingulate, caudal and rostral middle frontal, lateral and medial orbitofrontal, and the middle temporal (10,22,23). In addition, the middle temporal gyrus was chosen given its association with genetically predicted elevated IL-6 in the population (57). The eighth ROI included the volume of the nucleus accumbens, associated with the neurobiology of motivation, reward, and pleasure (58,59).

#### Circulating Levels of Plasma IL-6

For the BeneMin study, plasma levels of IL-6 were measured using V-PLEX Plus Proinflammatory Panel 1 Human Kit, and plates were read on using a Meso Scale Discovery QuickPlex SQ 120 and the Discovery Workbench software (45).

For the iRELATE study, plasma levels of IL-6 were measured using a quantikine high-sensitive enzyme-linked immunosorbent assay (Bio-Techne Catalog No. HS600C) (48).

### Statistical Analyses

#### Sociodemographic, Clinical, General Cognitive Performance, Plasma IL-6 Levels, and Structural Brain Metrics

Demographic and clinical data were analyzed using descriptive statistics according to data distribution. Categorical data were analyzed using Pearson’s χ^2^ or Fisher’s exact tests. Nonparametric data were analyzed using Mann-Whitney *U* tests.

To account for the effect of different assay methods on plasma IL-6 levels, the data were natural log-transformed and *z*-scored, and differences between groups were tested using GLMs. Both unadjusted and adjusted models (adjusted for sex, age, and chlorpromazine equivalent dose) were performed.

Structural brain metrics were analyzed using GLMs adjusted for sex, age, and chlorpromazine equivalent dose.

Results were considered significant at *p* < .05 (2-sided).

### GLMs for Associations Between Plasma IL-6 Levels and Depressive and Negative Symptoms

Using GLMs, we assessed associations between plasma IL-6 levels and the outcomes of PANSS depressive and negative symptom severity in individuals with schizophrenia.

Sample size and analytic models for these GLM analyses are summarized in Figure 1A, B and Table S2.

These analyses were conducted in 3 groupings as follows: 1) combined cohorts (*n* = 295 for PANSS-depression and *n* = 294 for PANSS-negative), 2) individuals with early schizophrenia (*n* = 201 for PANSS-depression and PANSS-negative), and 3) individuals with established schizophrenia (*n* = 94 for PANSS-depression and *n* = 93 for PANSS-negative).

For all analyses across the 3 groupings, plasma IL-6 levels were natural log-transformed and *z*-scored. Both unadjusted and adjusted models were performed, the latter including age, sex, and chlorpromazine equivalent dose as covariates. We did not adjust the results for body mass index (BMI) following evidence from recent literature working with the immunometabolic concept (60, 61, 62, 63), where metabolic factors, such as BMI, were not merely confounders but integral components of the inflammatory profile and immune dysregulation. Therefore, adjusting analyses for BMI would likely diminish and mask the inflammatory signal that is central to our investigation.

#### Sensitivity Analyses

In the iRELATE study, 18 individuals with schizophrenia had an illness duration ≤ 5 years. Therefore, we reran the GLM analyses excluding these 18 individuals. Analyses were adjusted for age, sex, and chlorpromazine equivalent dose.

### Structural Equation Modeling

An overview of sample size and analytic models for the SEM analyses is summarized in Figure 1C, D and Table S3.

We used SEM (64) in lavaan (version 0.6-12) (65) for R version 2022.07.02 (66) to examine the unique and combined relationships between plasma IL-6 levels, general cognitive performance, and structural brain metrics (CT and volume) and the severity of depressive and negative symptoms. First, we performed path analyses with composite scores (67) in the combined cohorts of individuals with early (*n* = 102) and established (*n* = 42) schizophrenia. Then we analyzed data from individuals with early schizophrenia only. We were precluded from performing separate path analyses with composite scores for individuals with established schizophrenia only because of the limited sample (*n* = 42) with complete data for plasma IL-6 levels, structural brain imaging, cognitive performance, and outcomes.

#### Calculation of Composite Scores for Plasma IL-6, Structural Brain Metrics, and General Cognitive Performance

Before running SEM, we performed confirmatory factor analysis (CFA) to calculate composite scores for 3 variables of interest: plasma IL-6 (plasma IL-6 levels), structural brain metrics (7 ROIs for brain CT and 1 ROI for volume), and general cognitive performance (verbal and performance IQ estimates). Factor scores for all composite scores derived from CFA were calculated using the factor score regression approach for 1-factor models (68,69), which were entered into the path analyses with composite scores in the next step. See Supplement section 3.3.1 for information on CFA analyses and model fit statistics.

#### Integrative Models

We ran 2 separate integrative models (PANSS depression and PANSS negative models) (Figure 1B). For both models, evaluated separately, we assessed whether higher plasma IL-6 levels (direct path) or reduced structural brain metrics (CT and volume; indirect path) would be associated with greater severity of depressive or negative symptoms.

For both models, the composite scores for plasma IL-6, structural brain metrics, and general cognitive performance were allowed to covary (70,71). Both unadjusted and adjusted models were estimated, the latter including sex, age, and chlorpromazine equivalent dose as covariates. See Supplement section 3.3.2 for chlorpromazine equivalent dose estimation. SEM results were not adjusted for BMI because this likely would have diminished and masked the inflammatory signal that is central to our investigation. The unadjusted models are presented in the Supplement.

## Results

### Early and Established Schizophrenia Samples for GLMs

#### Sociodemographic and Clinical Characteristics, General Cognitive Performance Measure, and Plasma IL-6 Levels

The characteristics of individuals with early and established schizophrenia for GLM analyses are detailed in Table S4. Individuals with early schizophrenia were significantly younger (*p* < .001) and had fewer years of education (*p* < .001) and lower BMIs (*p* < .001) compared with individuals with established schizophrenia, with no difference for sex (*p* = .313). In addition, individuals with early schizophrenia had significantly lower PANSS general symptom severity scores (*p* < .001) and slightly lower depressive symptom severity scores (*p* = .040). Mean values of chlorpromazine equivalent dose (*p* < .001) and plasma IL-6 levels (*p* < .001, unadjusted and adjusted for covariates) were significantly lower in the early schizophrenia group than in the established schizophrenia group. However, the 2 groups did not differ in general cognitive performance (*p* = .120).

#### Effect of Plasma IL-6 Levels on Depressive and Negative Symptom Severity in Early and Established Schizophrenia: GLMs

Both unadjusted and adjusted models are presented in Table 1, Table 2, Table 3. Below, we report results for GLMs adjusted for sex, age, and chlorpromazine equivalent dose.

**Table 1:** Effect of Plasma IL-6 Levels on the Severity of Depressive and Negative Symptoms in Early (BeneMin Sample) and Established (iRELATE Sample) Schizophrenia Combined

Outcome	*n*	Exposure	*B* (95% CI)		*p* Value	
			Unadjusted	Adjusted^a^	Unadjusted	Adjusted^a^
PANSS Depression	295	Plasma IL-6	0.76 (0.35 to 1.76)	0.82 (0.29 to 1.35)	<.001^b^	.002^b^
PANSS Negative	294	Plasma IL-6	0.23 (−0.32 to 0.78)	0.78 (0.14 to 1.41)	.416	.017^b^

Plasma IL-6 levels were natural log-transformed before standardization (*z*-transformed).

BeneMin, Benefit of Minocycline on Negative Symptoms of Psychosis: Extent and Mechanism; IL-6, interleukin 6; iRELATE, Immune Response & Social Cognition in Schizophrenia; PANSS, Positive and Negative Syndrome Scale.

a Adjusted for age, sex, and chlorpromazine equivalent dose (imputed measure).

b Significant results (*p* < .05).

**Table 2:** Effect of Plasma IL-6 Levels on the Severity of Depressive and Negative Symptoms in Early Schizophrenia Only (BeneMin Sample)

Outcome	*n*	Exposure	*B* (95% CI)		*p* Value	
			Unadjusted	Adjusted^a^	Unadjusted	Adjusted^a^
PANSS Depression	201	Plasma IL-6	0.89 (0.27 to 1.50)	0.89 (0.25 to 1.52)	.005^b^	.006^b^
PANSS Negative	201	Plasma IL-6	0.06 (−0.70 to 0.83)	0.34 (−0.47 to 1.14)	.872	.415

Plasma IL-6 levels were natural log-transformed before standardization (*z*-transformed).

BeneMin, Benefit of Minocycline on Negative Symptoms of Psychosis: Extent and Mechanism; IL-6, interleukin 6; PANSS, Positive and Negative Syndrome Scale.

a Adjusted for age, sex, and chlorpromazine equivalent dose (imputed measure).

b Significant results (*p* < .05).

**Table 3:** Effect of Plasma IL-6 Levels on the Severity of Depressive and Negative Symptoms in Established Schizophrenia Only (iRELATE Sample)

Outcome	*n*	Exposure	*B* (95% CI)		*p* Value	
			Unadjusted	Adjusted^a^	Unadjusted	Adjusted^a^
PANSS Depression	94	Plasma IL-6	0.58 (−0.08 to 1.25)	0.49 (−0.22 to 1.20)	.087	.178
PANSS Negative	93	Plasma IL-6	1.05 (0.35 to 1.75)	1.15 (0.41 to 1.89)	.003^b^	.002^b^

Plasma IL-6 levels were natural log-transformed before standardization (*z*-transformed).

IL-6, interleukin 6; iRELATE, Immune Response & Social Cognition in Schizophrenia; PANSS, Positive and Negative Syndrome Scale.

a Adjusted for age, sex, and chlorpromazine equivalent dose (imputed measure).

b Significant results (*p* < .05).

#### Effect of Plasma IL-6 Levels on Depressive and Negative Symptom Severity in Early and Established Schizophrenia: GLMs—PANSS Depression Model

In individuals with early and established schizophrenia combined, higher levels of plasma IL-6 were significantly related to more severe depressive symptoms (*B* = 0.82; 95% CI, 0.29 to 1.35; *p* = .002) (Table 1). However, when tested separately, higher levels of plasma IL-6 were significantly related only to more severe depressive symptoms in those with early schizophrenia (*B* = 0.89; 95% CI, 0.25 to 1.52; *p* = .006) (Table 2), but not in established schizophrenia (*B* = 0.49; 95% CI, −0.22 to 1.20; *p* = .178) (Table 3).

Moderation analysis adjusted for sex, age, and chlorpromazine equivalent dose revealed a significant group by plasma IL-6 levels interaction (*B* = 1.21; 95% CI, 0.06 to 2.35; *p* = .039), indicating that the association between plasma IL-6 levels on the severity of depressive symptoms varied as a function of group. The significant interaction validates our subgroup analyses of elevated plasma IL-6 levels associated with the severity of depressive symptoms in individuals with early schizophrenia only, but not in those with established schizophrenia.

#### Effect of Plasma IL-6 Levels on Depressive and Negative Symptom Severity in Early and Established Schizophrenia: GLMs—PANSS Negative Model

Similar to the PANSS depression model, combining both groups showed that higher levels of plasma IL-6 were significantly related to more negative symptoms (*B* = 0.78; 95% CI, 0.14 to 1.41; *p* = .017) (Table 1). However, when tested separately, higher levels of plasma IL-6 were significantly related only to more severe negative symptoms in those with established schizophrenia (*B* = 1.15; 95% CI, 0.41 to 1.89; *p* = .002) (Table 3), but not in those with early schizophrenia (*B* = 0.34; 95% CI, −0.47 to 1.14; *p* = .415) (Table 2).

Moderation analysis adjusted for sex, age, and chlorpromazine equivalent dose revealed no significant group by plasma IL-6 levels interaction (*B* = −0.64; 95% CI, −2.30 to 1.02; *p* = .457). Therefore, we acknowledge that our subgroup analyses for negative symptoms need to be carefully interpreted given the absence of significant interaction between group and plasma IL-6 levels.

#### Sensitivity Analyses

After removing 18 individuals with an illness duration equal or beyond 5 years in the iRELATE study, the results remained comparable, suggesting that the 18 participants were not influencing the findings. See Supplement section 4.1.3 for complete description of results.

### Early and Established Schizophrenia Samples for SEM

#### Sociodemographic, Clinical, General Cognitive Performance Measure, and Plasma IL-6 Levels

The characteristics of individuals in the early and established phases of schizophrenia for SEM analyses are detailed in Table 4. Findings were similar to those described for the GLM samples. However, in the SEM sample, only the severity of general symptoms measured by PANSS was significantly lower in the early schizophrenia group than the established schizophrenia group (*p* = .042). See Supplement section 4.2.1 for further description.

**Table 4:** Sociodemographic, Clinical, General Cognitive Performance Measure, and Plasma IL-6 Levels—Early (BeneMin) and Established (iRELATE) Schizophrenia Samples for Structural Equation Modeling

Variables	Early Schizophrenia, *n* = 102	Established Schizophrenia, *n* = 42	*p* Value
Demographic Characteristics			
Male^a^	78 (76.5%)	29 (69.0%)	.403
Age^b^, Years	25.3 (4.9)	44.7 (10.3)	<.001^c^
Education^b^^,^^d^, Years	13.8 (2.1)	15.1 (3.0)	.003^c^
Body Mass Index^b^^,^^e^	27.7 (7.4)	29.7 (4.8)	.011^c^

Clinical Characteristics			
Diagnosis^a^			
Schizophrenia	94 (92.2%)	28 (66.7%)	<.001^c^
Schizophreniform	1 (1.0%)	–	–
Psychotic disorder not otherwise specified	2 (1.9%)	–	–
Delusional disorder	1 (1.0%)	–	–
Schizoaffective disorder	4 (3.9%)	14 (33.3%)	<.001^c^
Illness Information^f^			
Illness onset age, years	–	28.4 (9.4)	–
Illness duration, years, median (minimum–maximum)	–	18.1 (1–40)	–
Illness duration, ≤5 years^g^	102 (100%)	6 (15.0%)	<.001^c^
PANSS^b^			
Positive	9.9 (4.4)	8.5 (2.2)	.067
Negative	9.9 (5.5)	9.2 (3.4)	.539
General^h^	17.7 (7.1)	20.1 (3.9)	.042^c^
Depressive	5.7 (3.7)	6.3 (2.2)	.317
Total^i^	37.5 (12.8)	37.8 (7.8)	.891
Antipsychotic Treatment^b^			
Chlorpromazine equivalent dose, mg^j^	348.5 (178.9)	873.8 (2203.1)	.128
Chlorpromazine equivalent dose, imputed, mg	377.5 (387.2)	907.4 (2018.5)	.016^c^
Depression Symptoms			
Hamilton Depression Rating Scale	–	4.0 (4.0)	–
Calgary Depression Scale for Schizophrenia	4.8 (4.3)	–	–
General Cognitive Performance Measure^b^^,^^k^			
Verbal IQ score	95.2 (18.0)	97.9 (18.7)	.576
Performance IQ score	87.0 (13.8)	87.4 (16.3)	.968
General cognitive performance as sum of verbal and performance IQ scores (Full Scale IQ score)	90.7 (14.6)	92.6 (17.1)	.689
Cytokine Levels, pg/mL			
Plasma IL-6, raw, unadjusted model, median (IQR)^l^	0.5 (0.4–1.0)	2.3 (1.8–3.6)	<.001^c^
Plasma IL-6, raw, adjusted model, median (IQR)^m^	0.5 (0.4–1.0)	2.3 (1.8–3.6)	.041^c^

Values are presented as mean (SD) or *n* (%) unless indicated otherwise.

BeneMin, Benefit of Minocycline on Negative Symptoms of Psychosis: Extent and Mechanism; GLM, generalized linear model; IL-6, interleukin 6; iRELATE, Immune Response & Social Cognition in Schizophrenia; PANSS, Positive and Negative Syndrome Scale.

a Pearson’s χ^2^ test.

b Mann-Whitney *U* test.

c Significant results (*p* < .05).

d Missing data: years of education (early schizophrenia, *n* = 38; established schizophrenia, *n* = 4).

e Missing data: body mass index (early schizophrenia, *n* = 1; established schizophrenia, *n* = 1).

f Missing data: illness onset age (established schizophrenia, *n* = 3); illness duration (established schizophrenia, *n* = 2).

g Fisher’s exact test (used for comparisons of categorical data when expected frequencies in contingency tables were less than 5).

h Missing data: PANSS general (early schizophrenia, *n* = 1).

i Missing data: PANSS total (early schizophrenia, *n* = 1).

j Missing data: chlorpromazine equivalent dose (early schizophrenia, *n* = 34; established schizophrenia, *n* = 7).

k Wechsler Adult Intelligence Scale-Third Edition (WAIS-III-R), shortened and prorated version of WAIS-III.

l GLM. IL-6 levels were natural log-transformed and *z*-scored, while the raw descriptive statistics are provided.

m GLM adjusted for age, sex, and chlorpromazine equivalent dose (imputed measure). IL-6 levels were natural log-transformed and *z*-scored, while the raw descriptive statistics are provided.

#### Structural Brain Metrics

GLM analyses showed that structural brain metrics for CT and volume did not differ between individuals with early and established schizophrenia (*p* > .05 for all) (Table 5).

**Table 5:** CAT12-Derived Structural Brain Measures for Metrics of Cortical Thickness and Volume Data—Early (BeneMin) and Established (iRELATE) Schizophrenia Samples for Structural Equation Modeling

Structural Brain Measures^a^	Early Schizophrenia, *n* = 102	Established Schizophrenia, *n* = 42	*p* Value
Cortical Thickness			
Caudal anterior cingulate	2.6 (0.2)	2.4 (0.2)	.412
Rostral anterior cingulate	2.7 (0.2)	2.6 (0.1)	.534
Caudal middle frontal	2.4 (0.2)	2.4 (0.1)	.168
Rostral middle frontal	2.3 (0.2)	2.3 (0.1)	.240
Lateral orbitofrontal	2.7 (0.1)	2.6 (0.1)	.076
Medial orbitofrontal	2.4 (0.2)	2.3 (0.1)	.324
Middle temporal	2.8 (0.2)	2.6 (0.1)	.453
Volume			
Nucleus accumbens	0.4 (0.1)	0.4 (0.1)	.224

Averaged right and left hemispheres are presented.

BeneMin, Benefit of Minocycline on Negative Symptoms of Psychosis: Extent and Mechanism; iRELATE, Immune Response & Social Cognition in Schizophrenia.

a Generalized linear model adjusted for age, sex, and chlorpromazine equivalent dose (imputed measure).

#### Calculation of Composite Scores for Plasma IL-6, Structural Brain Metrics, and General Cognitive Performance Using CFAs

CFA results are detailed in Supplement section 4.2.3.

#### Integrative Models

Below we describe findings for PANSS depression and negative models adjusted for sex, age, and chlorpromazine equivalent dose. The unadjusted models are presented in Figures S1A, B and S2A, B.

#### Integrative Models: Depressive and Negative Symptom Severity in Early and Established Schizophrenia Combined

**Figure 2:**
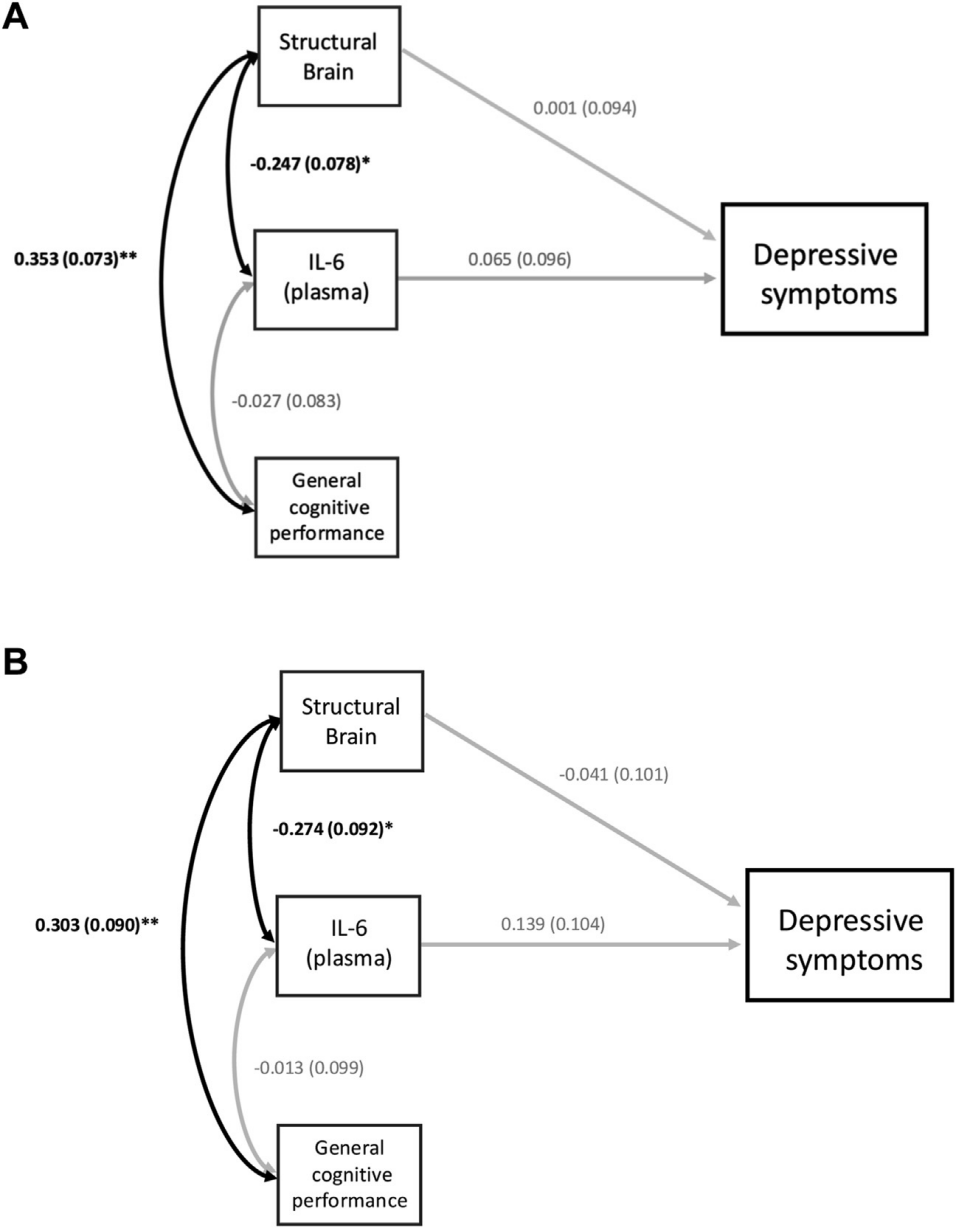
**(A)** Structural equation modeling (SEM)—early (BeneMin [Benefit of Minocycline on Negative Symptoms of Psychosis: Extent and Mechanism]) and established (iRELATE [Immune Response & Social Cognition in Schizophrenia]) samples combined. Positive and Negative Syndrome Scale-depression model, adjusted for age, sex, and chlorpromazine equivalent dose (imputed measure). Individuals with early schizophrenia (*n* = 102) and established schizophrenia (*n* = 42) combined. Estimates are standardized path coefficient composite scores. Single-headed arrows represent composite regression paths. Double-headed arrows depict covariances between the 3 endogenous variables. Black bold arrows denote significant associations (∗*p* < .05; ∗∗*p* < .001). Gray arrows represent nonsignificant associations. Factor score regression was used to generate composite scores for analyses. Confirmatory factor analysis (CFA) fit indices: χ^2^ = 0; comparative fix index (CFI) = 1; Tucker-Lewis index (TLI) = 1; root mean squared error of approximation (RMSEA) = 0; standardized root mean square residual (SRMR) = 0. SEM fit indices: χ^2^ = 5.972; CFI = 1; TLI = 1.104; RMSEA = 0; SRMR = 0.03. **(B)** SEM: early schizophrenia only (BeneMin sample). Positive and Negative Syndrome Scale-depression model adjusted for age, sex, and chlorpromazine equivalent dose (imputed measure). Individuals with early schizophrenia only (*n* = 102). Estimates are standardized path coefficients composite scores. Single-headed arrows represent composite regression paths. Double-headed arrows depict covariances between the 3 endogenous variables. Black bold arrows denote significant associations (∗*p* < .05; ∗∗*p* < .001). Gray arrows represent nonsignificant associations. Factor score regression was used to generate composite scores for analyses. CFA fit indices: χ^2^ = 0; CFI = 1; TLI = 1; RMSEA = 0; SRMR = 0. SEM fit indices: χ^2^ = 12.867; CFI = 0.830; TLI = 0.694; RMSEA = 0.053; SRMR = 0.055. IL-6, interleukin 6.

**Figure 3:**
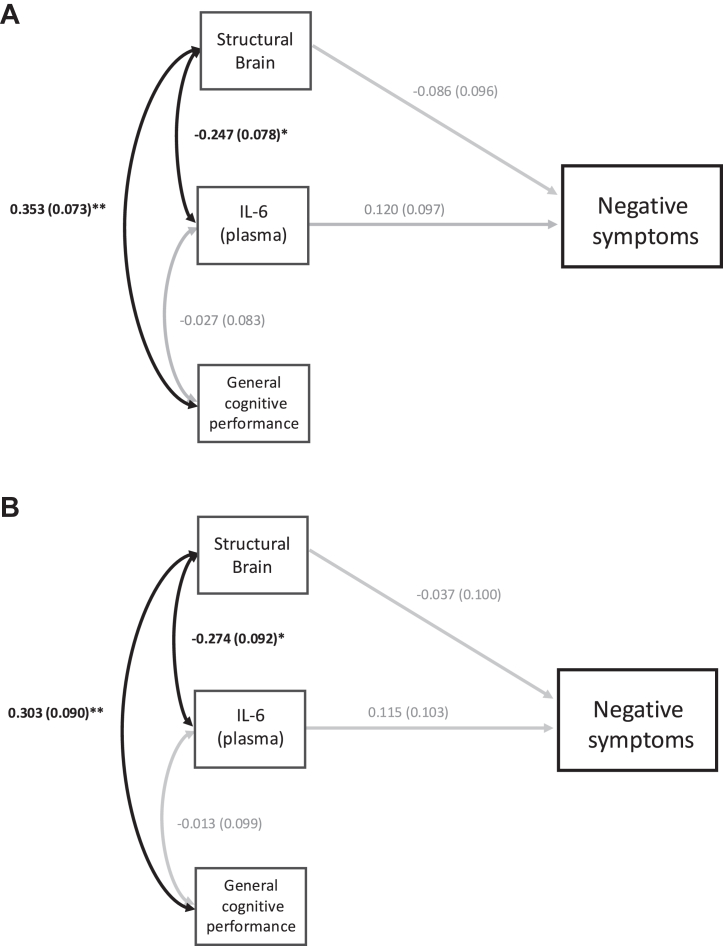
**(A)** Structural equation modeling (SEM): early (BeneMin [Benefit of Minocycline on Negative Symptoms of Psychosis: Extent and Mechanism]) and established (iRELATE [Immune Response & Social Cognition in Schizophrenia]) samples combined. Positive and Negative Syndrome Scale-negative model adjusted for age, sex, and chlorpromazine equivalent dose (imputed measure). Individuals with early (*n* = 102) and established schizophrenia (*n* = 42) combined. Estimates are standardized path coefficient composite scores. Single-headed arrows represent composite regression paths. Double-headed arrows depict covariances between the 3 endogenous variables. Black bold arrows denote significant associations (∗*p* < .05; ∗∗*p* < .001). Gray arrows represent nonsignificant associations. Factor score regression was used to generate composite scores for analyses. Confirmatory factor analysis (CFA) fit indices: χ^2^ = 0; comparative fit index (CFI) = 1; Tucker-Lewis index (TLI) = 1; root mean squared error of approximation (RMSEA) = 0; standardized root mean square residual (SRMR) = 0. SEM fit indices: χ^2^ = 12.463; CFI = 0.954; TLI = 0.892; RMSEA = 0.052; SRMR = 0.044. **(B)** SEM: early schizophrenia (BeneMin) sample only. Positive and Negative Syndrome Scale-negative model adjusted for age, sex, and chlorpromazine equivalent dose (imputed measure). Individuals with early schizophrenia only (*n* = 102). Estimates are standardized path coefficients composite scores. Single-headed arrows represent composite regression paths. Double-headed arrows depict covariances between the 3 endogenous variables. Black bold arrows denote significant associations (∗*p* < .05; ∗∗*p* < .001). Gray arrows represent nonsignificant associations. Factor score regression was used to generate composite scores for analyses. CFA fit indices: χ^2^ = 0; CFI = 1; TLI = 1; RMSEA = 0; SRMR = 0. SEM fit indices: χ^2^ = 16.127; CFI = 0.726; TLI = 0.508; RMSEA = 0.078; SRMR = 0.059. IL-6, interleukin 6.

SEM results and full statistics are presented in Figures 2A and 3A and Tables S6A and S7A.

In the combined sample, neither the plasma IL-6 composite score nor the structural brain metrics composite score predicted depressive or negative symptom severity (*p* > .05 for both).

For both PANSS-depression and -negative models, among the 3 composite scores derived from CFA, the general cognitive performance composite score was significantly and positively related to the structural brain metrics composite score (*p* < .05), while the plasma IL-6 composite score was significantly and negatively related to the structural brain metrics composite score (*p* < .05). Finally, the plasma IL-6 composite score was unrelated to the general cognitive performance composite score (*p* > .05).

#### Integrative Models: Depressive and Negative Symptom Severity in Early Schizophrenia Only

SEM results and full statistics are presented in Figure 2, Figure 3 and Tables S6B and S7B.

Neither the plasma IL-6 composite score nor the structural brain metrics composite score predicted the severity of depressive symptoms (*p* > .05 for both).

For both PANSS-depression and -negative models, the general cognitive performance composite score was significantly and positively related to the structural brain metrics composite score (*p* < .05). The plasma IL-6 composite score was significantly and negatively related to the structural brain metrics composite score (*p* < .05), but the plasma IL-6 composite score was unrelated to the general cognitive performance composite score (*p* > .05).

## Discussion

To our knowledge, this is the first study to evaluate associations between higher plasma IL-6 levels and depressive and negative symptom severity in individuals with schizophrenia and explore the role of illness stage. We found that higher plasma IL-6 levels were significantly related to more severe depressive symptoms in early schizophrenia and more severe negative symptoms in established schizophrenia. However, the association between higher IL-6 levels and the severity of negative symptoms in established schizophrenia was observed in the absence of a moderation effect; therefore, the findings should be interpreted carefully. These findings partially support our first hypothesis that elevated plasma IL-6 levels would be related to greater depressive and negative symptom severity in schizophrenia. Using SEM, we found that the interrelationships between higher plasma IL-6 levels, structural brain metrics, and general cognitive performance were not associated with greater depressive or negative symptom severity. However, plasma IL-6 levels were significantly associated with reduced structural brain metrics, even though reduced structural brain metrics did not predict the severity of depressive and negative symptoms directly. We also did not observe any significant associations between plasma IL-6 levels and general cognitive performance, whereas we found a significant relationship between lower CT and volume with general cognitive performance. These findings did not support our second hypothesis.

### GLM Findings

The GLM analyses demonstrated significant associations between higher plasma IL-6 levels (natural log and *z*-scored) and more severe depressive symptoms in early schizophrenia and negative symptoms in established schizophrenia (the latter in the absence of a significant moderation effect).

Previous evidence suggests that higher peripheral IL-6 levels are associated with more severe depressive symptoms in early-stage schizophrenia, particularly in drug-naïve individuals with first-episode psychosis (72), although conflicting findings exist for studies conducted with medicated individuals with early-stage schizophrenia (8,73,74). Regarding the association between higher peripheral IL-6 levels and negative symptoms during the early stages of the illness, a systematic review of 6 studies of drug-naïve individuals with first-episode psychosis (75) showed that only 1 study found a significant correlation between peripheral IL-6 levels and more severe negative symptoms (76). One additional study did not observe significant associations between peripheral IL-6 levels and 2 subdomains of negative symptoms at multiple time points (8), supporting our findings.

In individuals with established schizophrenia, some evidence suggests that negative symptoms rather than depressive symptoms may be related to higher peripheral IL-6 levels. Goldsmith *et al.* (77) found higher peripheral IL-6 levels in individuals with established schizophrenia with a deficit syndrome marked by primary and enduring negative symptoms compared with individuals without this syndrome and healthy control participants. Additional support comes from a study that showed that higher peripheral IL-6 levels were related to more severe negative symptoms (78) in a subgroup of individuals identified as so-called nonresponders to antipsychotic treatment, but not in individuals who were identified as responders to antipsychotic treatment. Additional studies are needed to shed light on this hypothesized pattern in established schizophrenia.

We highlight that in our sample of individuals with early and established schizophrenia, negative and depressive symptom severity were unrelated. Thus, distinct mechanisms may be at play. While our assumptions are speculative and should be interpreted with caution, they may indicate novel avenues to examine plasma IL-6 levels depending on the illness phase.

### Integrative SEM Findings

Next, we examined potential neurobiological and neurocognitive mechanisms by assessing the unique and combined relationships between plasma IL-6 levels, general cognitive performance, and structural brain metrics. In the combined sample of early and established schizophrenia and in early schizophrenia only, the interrelationship between higher plasma IL-6 levels, structural brain metrics, and general cognitive performance did not significantly predict more severe depressive or negative symptoms. Our findings could be that our sample size may be too small for SEM analyses despite our factor score regression approach, which has been proposed to overcome such small sample sizes (68). In addition, the potentially limited variance in psychopathology, particularly the PANSS scores, which were indicative of relatively mild negative and depressive symptom scores in our cohorts, might have influenced our findings. The absence of individuals with scores indicative of more severe symptoms (typically above 15–20 on the PANSS scale) (79) likely reduced our ability to detect significant associations in SEM. Future studies should consider including participants with a wider range of symptom severity to better capture potential relationships between IL-6, brain structure, and clinical outcomes. Alternatively, it may be that the relationship between plasma IL-6 levels and depressive and negative symptoms may involve other or additional pathways that were not evaluated in our study, such as childhood maltreatment, which is related to low-grade inflammation (80, 81, 82), cognitive deficits (83, 84, 85), reduced structural (49,86,87) and functional (47,88) brain metrics, and poor clinical outcomes (89).

Previous studies linking elevated peripheral IL-6 with reduced CT and brain volumes focused on established schizophrenia without considering antipsychotic effects, while our findings, consistent with a UK Biobank Mendelian randomization study (57), suggest that genetically predicted IL-6 levels are associated with reduced brain CT and volumes, particularly in the middle temporal gyrus, although no significant association with cognitive performance was found in our models, consistent with previous meta-analyses (39,40). We discuss these findings more in Supplement section 5.

### Strengths and Limitations

Our study has several strengths. We applied SEM to assess the relationships of plasma IL-6 levels, CT and volume of 8 ROIs, and general cognitive performance to depressive and negative symptom severity in individuals with early and established schizophrenia. This approach has the advantage of examining the complex interplay among biological and psychological measures. However, we acknowledge that our sample size may be small for SEM analyses, even though we used the factor score regression approach to overcome this limitation. Nevertheless, this method has some drawbacks (68,69). In addition, our models (SEM) are equal to the recommended guideline of *n* > 5 per estimated path (90). Another limitation is the relatively mild severity of negative and depressive symptoms that was observed in our cohorts. This potentially limited variance in symptom severity might have reduced the sensitivity of our analyses to detect significant associations, particularly in SEM. Additionally, different assay methods were used to measure plasma IL-6 levels (V-Plex and enzyme-linked immunosorbent assay) in the 2 cohorts. In the iRELATE study, blood samples were collected at approximately the same time of day (9:30 am); however, information about time of blood draw is not available for the BeneMin study. Furthermore, we lack detailed information regarding the duration of freezer storage prior to IL-6 assay measurement in both cohorts. To mitigate potential differences, data on plasma IL-6 levels were natural log-transformed and *z*-scored. Given our null SEM results and sample size, we did not adjust for scanner type or site to avoid overfitting of the models. Moreover, the coordination of MRI sequences across sites in the BeneMin cohort was guided by the NeuroPsyGrid (91) multicenter validation procedure study, and a calibration study was conducted prior to the investigation to minimize scanner variability (see the Supplement). We acknowledge that future research should aim to investigate IL-6 levels alongside other cytokines to provide a more integrative view of the inflammatory status. We do not discount the possible influence of comorbidities or inflammatory conditions on plasma IL-6 levels given that these were not exclusion criteria in either cohort. Regarding adjustment for BMI, tobacco smoking, and cannabis use, previous meta-analyses of individuals with recent and chronic psychotic disorders have shown elevated plasma IL-6 levels after adjusting for BMI and tobacco smoking (28,32). Our previous study demonstrated that daily cannabis use was not associated with increased inflammation (92). Finally, our findings are not directly comparable to studies that have investigated cerebrospinal fluid IL-6 levels in schizophrenia, which have not addressed an association with negative and depressive symptoms (93). Future studies that incorporate blood and cerebrospinal fluid measurements from matched individuals will be crucial to validating our findings and providing a more comprehensive understanding of the role of IL-6 in the central nervous system in schizophrenia.

### Conclusions

Our results indicate that higher plasma IL-6 levels may be differently associated with the severity of depressive and negative symptoms in schizophrenia depending on the illness stage. Associations between higher plasma IL-6 levels and the severity of depressive and negative symptoms may occur independently of their relationship with lower general cognitive performance and reduced structural brain metrics. Understanding the relationship between plasma IL-6 levels and depressive and negative symptoms may help unravel the heterogeneity of schizophrenia, paving the way for biologically informed substratification of patients in future experimental clinical research. Future studies should identify individuals with psychosis who have elevated levels of inflammation.

## Supplementary Material

Supplement Methods, Results, Discussion, Figures S1–S2, and Tables S1–S7

Key Resources Table
