# Supplementary material for: Depressive and Negative Symptoms in the Early and Established Stages of Schizophrenia: Integrating Structural Brain Alterations, Cognitive Performance, and Plasma Interleukin 6 Levels
Source: Biol Psychiatry Glob Open Sci. 2024 Dec 2;5(2):100429. doi: 10.1016/j.bpsgos.2024.100429 (PMC11795630; doi:10.1016/j.bpsgos.2024.100429)
Supplement: Supplement Methods, Results, Discussion, Figures S1–S2, and Tables S1–S7 [file mmc1.pdf]

SUPPLEMENTARY INFORMATION

Depressive and Negative Symptoms in the Early and Established Stages of Schizophrenia: Integrating Structural Brain Alterations, Cognitive Performance, and Plasma Interleukin 6 Levels

Corsi-Zuelli *et al.*

2. Materials and Methods

2.1 Participants

2.1.1 BeneMin study

BeneMin was a double-blind, randomized controlled trial testing the potential benefit of the anti-inflammatory minocycline on negative symptoms and cognition in individuals in an acute episode of psychosis (schizophrenia, schizophreniform, or schizoaffective psychosis). Two hundred and seven participants aged 16-35 years were recruited from eleven UK NHS trusts and met the criteria for schizophrenia spectrum disorder evaluated using the Mini-International Neuropsychiatric Interview (MINI) (1). All individuals were within the five years of their first presentation to mental health services, taking stable antipsychotic treatment, and were assisted by the UK NHS Early Intervention Services. In addition, all individuals had mild persisting psychotic symptoms evaluated by the Positive and Negative Syndrome Scale (PANSS) (2), considering a score of greater than two on the delusions, hallucinations, suspiciousness, or disorganization items. Participants who were significantly impacted by alcohol or substance abuse, experienced difficulties in communicating fluently in English, had a premorbid Intelligence Quotient (IQ) of less than 70 or were at risk of suicide or violence were excluded from the study. The study was approved by the North West Research Ethics Committee (ref.11/NW/0218), and all the participants provided written informed consent.

2.1.2 iRELATE study

We included data from individuals with Established schizophrenia from the "Immune Response & Social Cognition in Schizophrenia", iRELATE study. One hundred and four individuals aged between 18 and 65 years were recruited from local outpatient services in Dublin and Galway, Ireland. (3–5). Individuals with schizophrenia from the iRELATE study were classified as chronic schizophrenia if duration of illness was 12 months or above and clinically stable at the time of assessment – hereafter called Established schizophrenia.

iRELATE aimed to investigate the impact of environment, genes, and the immune system on brain structure and function in schizophrenia funded by the European Research Council. The following inclusion criteria were considered for individuals with schizophrenia: a) a diagnosis of schizophrenia confirmed by the Structured Clinical Interview (SCID) for Diagnostic and

Statistical Manual of Mental Disorders, 4th Edition (DSM-IV) (6); b) required to be clinically stable at the time of assessment; and c) absence of comorbid psychiatric disorders. Participants with schizophrenia were excluded if they had any of the following: a) a history of acquired brain injury leading to consciousness loss for longer than one minute; b) substance abuse in the previous six months; c) Intelligence Quotient (IQ) <70 indicating intellectual disability; d) neurological disorder (e.g., epilepsy); e) contra-indications for MRI or blood draws. The National University of Ireland Galway Research Ethics Committee, the Clinical Research Ethics Committee at University Hospital Galway and the Research Ethics Committee at Tallaght Hospital in Dublin reviewed and approved all study procedures. All the participants provided written informed consent.

2.2.1 Clinical assessment

The severity of positive, negative, and general psychopathology of schizophrenia was assessed using the PANSS scale (7). PANSS is a 30-item semi-structured interview containing three subscales, including the a) positive symptoms scale (PANSS-P; seven items); b) negative symptom scale (PANSS-N; seven items); and c) general psychopathology scale (PANSS-G; 16 items). The total scores for each subscale were: PANSS-P and PANSS-N (7 to 49), PANSS-G (16 to 112), and for PANSS total score 30 to 210.

2.2.1.1 Assessment of positive symptoms

Positive symptoms were measured using the PANSS-P subscale consisting of seven items (PANSS-P1: delusions; PANSS-P2: conceptual disorganization; PANSS-P3: hallucinatory behavior; PANSS-P4: excitement; PANSS-P5: grandiosity; PANSS-P6: suspiciousness/persecution; PANSS-P7: hostility).

2.2.1.2 Assessment of negative symptoms

Negative symptoms were measured using the PANSS-N subscale consisting of seven items (PANSS-N1: blunted affect; PANSS-N2: emotional withdrawal; PANSS-N3: poor rapport; PANSS-N4: passive/apathetic social withdrawal; PANSS-N5: difficulty in abstract thinking; PANSS-N6: lack of spontaneity and flow of conversation; PANSS-N7: stereotyped thinking).

2.2.1.4 General psychopathology scale

PANSS general psychopathology scale (PANSS-G) consists of 16 items (PANSS-G1: somatic concern; PANSS-G2: anxiety; PANSS-G3: guilty feelings; PANSS-G4: tension; PANSS-G5: mannerism and posturing; PANSS-G6: depression; PANSS-G7: motor retardation; PANSS-G8: uncooperativeness; PANSS-G9: unusual thought content; PANSS-G10: disorientation; PANSS-G11: poor attention; PANSS-G12: lack of judgment and insight; PANSS-G13: disturbing of volition; PANSS-G14: poor impulse control; PANSS-G15: preoccupation; PANSS-G16: active social avoidance.

2.2.2 General cognitive performance

The shortened version of WAIS-III is commonly used to reduce the time of administration and is highly correlated (r>0.9) with the full version containing eleven subsets (8,9). The pro-rated version was used in both studies and according to previous publications (10,11).

2.2.3 Magnetic Resonance Imaging

2.2.3.1 Magnetic Resonance Imaging acquisition and image processing

2.2.3.1.1 BeneMin study

Structural Magnetic Resonance Imaging (MRI) scans using 3 T MRI scanners were acquired at each study site. The sequences included three-dimensional T1-weighted magnetisation-prepared rapid gradient-echo (MPRAGE/SPGR), as detailed in (12) and the BeneMin study protocol (13).

The coordination of MRI sequences across imaging centres was based on the previous NeuroPsygGrid multi-centre validation and reliability study (14). As detailed in the study protocol (13), MRI data were gathered from six MRI centres using six 3T MRI scanners, including two Philips Achieva (Philips Medical Systems, Best, Netherlands), two Siemens Trim Trio, one Siemens Verio (Siemens Medical Systems, Erlangen, Germany), and one GE Signa (General Electric, Milwaukee, WI, USA). As there were technical variations among the scanners, replicating the scanning parameters directly would not yield functionally equivalent MRI protocols. Therefore, to develop BeneMin MRI protocols, researchers have leveraged the

expertise Established by the Alzheimer's Disease Neuroimaging Initiative (ADNI), a multi-centre imaging project dedicated to standardising MRI across sites and manufacturers. Additionally, researchers conducted a calibration study before the trial. Please refer to (13) for further information.

Briefly, in the BeneMin study, the specifications for each sequence were as follows:
1) MPRAGE/SPGR: Voxel size of approximately $1 \times 1 \times 1$ mm^3, phase encode on the anterior-posterior plane, and whole brain recorded.
2) PD/T2: Voxel size of approximately $1 \times 1 \times 3$ mm^3, phase encode on the right-left plane, and whole brain recorded.
3) EPI: TR = 2,000 ms, 748 three-dimensional volumes, voxel size of approximately $3 \times 3 \times 4.5$ mm^3, slices aligned to AC-PC axis, phase encode on the anterior-posterior plane, and whole brain recorded.

2.2.3.1.2 iRELATE study

MRI data for the iRELATE study were obtained using a 3T Philips Achieva MR scanner (Philips Medical Systems, the Netherlands) located in the Centre for Advanced Medical Imaging at St James's Hospital, Dublin.

Brain MRI acquisition details were: three-dimensional T1-weighted structural scans (Fast Field Echo, FFE) pulse sequence, TR (Repetition Time)/TE (Echo Time) = 8.5/3.9 ms, FOV (Field-of-view) = 256 x 256 x 160 mm^3, a spatial resolution of 1 mm^3, TI (Inversion Time) = 1060 ms, flip angle = 8°, SENSE (Sensitivity Encoding) factor = 1.5 and acquisition time = 7 min 30 s) of the whole brain were obtained for each participant in the iRELATE study. Please, refer to (5) for further information.

2.2.3.2 Structural MRI analysis

Images from both BeneMin and iRELATE samples were processed using the open-source CAT12 toolbox (http://dbm.neuro.uni.jena.de/cat12/) within SPM12 (Statistical Parametric Mapping software, http://www.fil.ion.ucl.ac.uk/spm/) using MATLAB R2023B. We chose CAT-12 as an alternative for FreeSurfer for the assessment of CT and volume metrics due to its fast and user-friendly approach, as well as its demonstrated accuracy, robustness, and

equally performance in detecting atrophic brain areas, as evidenced by excellent test-retest variability scores in comparison studies (15,16).

Please refer to the CAT12 toolbox manual (http://www.neuro.uni-jena.de/cat12/CAT12-Manual.pdf) and (17) for comprehensive details on the MRI processing steps. Based on the standard CAT12 protocol, default parameter settings were adopted for processing and analyses. Briefly, all T1-weighted data underwent processing with CAT12, using the cross-sectional processing stream for voxel-based morphometry (VBM), surface-based morphometry (SBM; cortical thickness), and regions of interest (ROIs) analyses, as detailed in the CAT12 toolbox manual. For VBM, the T1-weighted images underwent segmentation into grey matter (GM), white matter (WM), and cerebrospinal fluid (CSF) using segmentation tools. Following this, the segmented GM images were spatially normalised to a template in Montreal Neurological Institute (MNI) space (1.5 mm cubic resolution in MNI space) using high-dimensional Diffeomorphic Anatomical Registration Through Exponentiated Lie Algebra (DARTEL). The processing stream yielded modulated and registered grey matter segments, which were smoothed with a 6 mm Gaussian kernel. Surface-based processing included segmentation, topological correction, spherical mapping, spherical registration, and CT estimation. SBM analysis resulted in registered point-wise cortical thickness measures, smoothed with a 12 mm Gaussian kernel. Voxel-based ROI analysis utilised the Neuromorphometrics atlas to compute regional grey matter volumes, while surface-based ROI analysis employed the DK40 atlas to calculate regional cortical thickness (18).

2.2.4 Circulating levels of plasma interleukin-6

2.2.4.1 BeneMin study

Venous blood samples at baseline were collected in 9 mL EDTA tubes and underwent centrifugation (2,000g for 5 minutes at room temperature) within four hours of collection. aliquots were stored at -80ºC for future analysis.

2.2.4.2 iRELATE study

Whole blood samples at baseline were collected in 6 mL EDTA tubes. All samples were obtained at approximately the same time of the day (9:30 am). The samples were centrifuged

at 1,200g for 10 minutes at room temperature. Then, plasma was aliquoted in 1.5mL Eppendorf tubes and kept at -80ºC for future analysis.

3. Statistical analyses

3.3.1 Calculation of composite scores plasma IL-6, structural brain metrics, and general cognitive performance

We ran Confirmatory Factor Analysis (CFA) to calculate composite scores for three variables of interest: 'plasma IL-6'; 'structural brain metrics'; and 'general cognitive performance'. Factor scores for all composite scores derived from CFA were calculated using the Factor Score Regression (FSR) approach for one-factor models (19,20), which were entered into the path analyses with composite scores in the next step.

The composite score 'plasma IL-6' was created using a single plasma IL-6 continuous variable, which reflects the circulating level of plasma IL-6 at baseline.

We calculated the 'structural brain metrics' composite score based on the seven ROIs for CT and one ROI for brain volume (please see Table S1 and item 2.2.3.3),

The composite score for 'general cognitive performance' was created based on IQ continuous variables (verbal and performance estimates) of the WAIS-III.

To assess model fit of CFA and SEM, we report chi-square (X^2) fit statistics, and the root mean squared error of approximation (RMSEA). RMSEA of less than 0.08 implies an acceptable model fit, and values of less than 0.05 imply a good fit (21). We also report the comparative fit index (CFI) and the Tucker-Lewis index (TLI), for which the values of CFI and TLI > 0.95 represent a good fit of the overall model (21), and the standardised root squared residual <0.08 (SMRM).

In the CFA, we note that all reported fit statistics and indices are over-identified. This means that our models were fully saturated and had a perfect fit with 0 degrees of freedom; therefore, we report and focus our discussion on the key associations within the models and will not interpret model fit indices. We employed the estimator maximum likelihood (ML) and used full-information maximum likelihood (FIML), which is optimal for models that contain missing data that are assumed to be missing at random (21,22). Using FIML, the estimation assesses all parameters based on the cases that have available data. All variables were scaled to a standard normal distribution.

3.3.2 Integrative models

Chlorpromazine equivalent dose was available for the iRELATE sample, whereas we converted the olanzapine equivalent dose to chlorpromazine equivalent using the chlorpromazineR package for R (https://github.com/ropensci/chlorpromazineR; https://cran.r-project.org/web/packages/chlorpromazineR/vignettes/walkthrough.html) for the BeneMin sample. Missing data for the chlorpromazine equivalent (iRELATE n=7; BeneMin n=34) were imputed using a fully conditional specification model with predictive mean matching (23) before running SEM.

4. Results

4.1 Early and Established schizophrenia samples for Generalized Linear Models

4.1.1 Socio-demographic, clinical, general cognitive performance measure, and plasma IL-6 levels

The severity of depressive and negative symptoms measured by PANSS was not correlated in Early (rho=0.080, p=0.252, n=206) or Established (rho=0.158, p=0.117, n=100) schizophrenia individuals tested separately or combined (rho=0.092, p=0.107, n=306).

In the Established schizophrenia group, 33.7% were on clozapine, while twelve (5.8%) were taking clozapine in the Early schizophrenia group.

4.1.3 Sensitivity analyses

After removing 18 individuals with Established schizophrenia from the iRELATE study, the results remained comparable.

Plasma IL-6 levels were still significantly higher in Established schizophrenia compared to individuals with Early schizophrenia (Established schizophrenia: raw plasma IL-6 levels, median = 2.17, Interquartile range (IQR) = 1.72 – 3.79); Early schizophrenia: median = 0.61, IQR = 0.39 – 0.96; p<0.001).

In the combined sample, higher plasma IL-6 levels were still significantly related to more severe depressive (B = 1.01; 95% CI = 0.41, 1.61; p<0.001) and negative symptoms (B = 0.91; 95% CI = 0.03, 1.79; p=0.044). In addition, higher levels of plasma IL-6 in individuals with Established schizophrenia were still significantly related to more severe negative (B = 1.36; 95% CI = 0.47, 2.23; p=0.002), but not depressive symptoms (B = 0.57; 95% CI = -0.11, 1.25; p = 0.097).

These results show that the 18 participants with an illness duration equal or beyond five years in the iRELATE study were not influencing the findings.

4.2 Early and Established schizophrenia samples for Structural Equation Modeling (SEM)

4.2.1 Socio-demographic, clinical, general cognitive performance measure, and plasma IL-6 levels

In individuals at the Established phase of schizophrenia, the severity of depressive and negative symptoms was moderately correlated (rho=0.344, p=0.026, n=42), but this correlation was not observed in individuals at the Early phase of schizophrenia (rho=0.005, p=0.960, n=102). When combining both samples, we found no relationship between the severity of depressive and negative symptoms (rho=0.065, p=0.440, n=144).

4.2.3 Calculation of composite scores for plasma IL-6, structural brain metrics, and general cognitive performance using Confirmatory Factor Analyses

4.2.3.1 Individuals with Early schizophrenia and Established schizophrenia combined

All composite scores loadings were positive when both samples were combined (Supplementary Table S5A). These results suggest that higher scores for plasma IL-6, structural brain metrics, and general cognitive performance measure reflect elevated levels of plasma IL-6, higher CT and volume of the ROIs and better general cognitive performance. Standardised estimates for relations among the three composite scores among the combined samples are shown in Table S6A (PANSS depression model) and Table S7A (PANSS negative model).

4.2.3.2 Individuals with Early schizophrenia only

All composite score loadings remained positive (Supplementary Table S5B). These results suggest that higher scores for plasma IL-6, structural brain metrics, and general cognitive performance measure reflect elevated levels of plasma IL-6, higher CT and volume of the ROIs and better general cognitive performance. Standardised estimates for relations among the three composite scores derived from CFA are shown in Supplementary Table S6B (PANSS depression model) and Table S7B (PANSS negative model).

5. Discussion

The relationship between higher plasma IL-6 levels and lower CT and volume was consistent in both SEM models evaluated in the combined cohorts and in Early schizophrenia only. Previous studies suggesting associations between elevated peripheral IL-6 levels with reduced CT and volumes of prefrontal and temporal regions were predominantly conducted in individuals with Established schizophrenia, without accounting for potential confounding by antipsychotic medication (24–26). Our findings are in keeping with one MR study using the UK Biobank showing that genetically predicted peripheral IL-6 levels were associated with reduced brain CT and volumes in the general population, particularly in the middle temporal gyrus, a region in which gene expression was enriched for IL-6 pathways and schizophrenia (27).

Finally, in our two SEM models, general cognitive performance was not significantly associated with plasma IL-6 levels. Inconsistent findings from recent meta-analyses show weak positive relationships between higher peripheral IL-6 levels and reduced global or specific cognitive domains (28,29) in mixed samples of individuals with schizophrenia. However, the studies were cross-sectional and did not consider illness stage. Specific mechanisms can plausibly be examined when subgroups of individuals with mildly elevated plasma IL-6 levels and circumscribed cognitive deficits are identified.

Table S1. Key function of selected regions of interest (ROIs)

ROIs	Function	References
Caudal and rostral anterior cingulate	Caudal: emotion regulation and error detection<br><br>Rostral: decision making, social cognition, and processing conflicts	(30–33)
Caudal and rostral middle frontal	Caudal: cognitive control and working memory<br><br>Rostral: higher level executive function and goal-directed behaviour	(31,32)
Lateral and medial orbitofrontal	Lateral: decision-making and reward processing<br><br>Medial: emotion regulation and social interactions	(30–32,34)
Middle temporal	Language processing, motion perception, and integration of sensory information for visual and auditory processing	(31,32)
Nucleus accumbens	Motivation, reward, reinforcement learning, pleasure, and addiction	(35)

Table S2. Models tested in Generalised Linear Model

Model	Study (n)	Sample	Exposure	Outcome
PANSS depression	BeneMin (201)	Early schizophrenia	Plasma IL-6 levels	PANSS depression scores
PANSS depression	iRELATE (94)	Established schizophrenia	Plasma IL-6 levels	PANSS depression scores
PANSS depression	BeneMin (201) and iRELATE (94) combined	Early and Established schizophrenia	Plasma IL-6 levels	PANSS depression scores
PANSS negative	BeneMin (201)	Early schizophrenia	Plasma IL-6 levels	PANSS negative scores
PANSS negative	iRELATE (93)	Established schizophrenia	Plasma IL-6 levels	PANSS negative scores
PANSS negative	BeneMin (201) and iRELATE (93) combined	Early and Established schizophrenia	Plasma IL-6 levels	PANSS negative scores

Both unadjusted and adjusted models were estimated, the latter including sex, age, and chlorpromazine equivalent dose.

Plasma IL-6 levels were natural log-transformed before standardisation (Z-transformed).

Table S3. Models tested in Structural Equation Modelling

Model	Study (n)	Sample	Composite scores derived from CFA	Outcome
PANSS depression	BeneMin (102)	Early schizophrenia	'plasma IL-6', structural brain', 'general cognitive performance'	PANSS depression scores
PANSS depression	BeneMin (102) and iRELATE (42) combined	Early and Established schizophrenia	'plasma IL-6', structural brain', 'general cognitive performance'	PANSS depression scores
PANSS negative	BeneMin (102)	Early schizophrenia	'plasma IL-6', structural brain', 'general cognitive performance'	PANSS negative scores
PANSS negative	BeneMin (102) and iRELATE (42) combined	Early and Established schizophrenia	'plasma IL-6', structural brain', 'general cognitive performance'	PANSS negative scores

Both unadjusted and adjusted models were estimated, the latter including sex, age, and chlorpromazine equivalent dose.

Table S4: Socio-demographic, clinical, general cognitive performance, and plasma IL-6 levels – Early (BeneMin) and Established schizophrenia (iRELATE) for GLM analyses

Variables	Early schizophrenia (n=207)*	Established schizophrenia (n=104)	p
**Demographic and clinical**			
Males, n (%)[1]	150 (72.8)	70 (67.3)	0.313
Age, mean (SD)[2#]	25.6 (5.1)	43.1 (11.0)	**<0.001**
Years of education, mean (SD)[2#]	13.8 (2.0)	14.9 (3.3)	**<0.001**
Body mass index (kg/m^2), mean (SD)[2#]	27.9 (6.9)	29.6 (5.1)	**<0.001**
**Diagnosis, n (%)**[1#]			
Schizophrenia	180 (87.4)	73 (70.2)	**<0.001**
Schizophreniform	4 (1.9)	-	
Psychotic disorder not otherwise specified	10 (4.9)	-	
Delusional disorder	1 (0.5)	-	
Schizoaffective disorder	7 (3.4)	30 (28.8)	**<0.001**
**Illness information#**			
Illness onset age, mean (SD)	-	27.3 (8.6)	-
Illness duration (years), median (min-max)	-	17.1 (1 - 40)	-
Illness duration, n % (≤ 5 years)[3]	206 (100)	18 (18.2)	**<0.001**
**PANSS, mean (SD)**[2#]			
Positive	9.9 (4.9)	8.9 (2.8)	0.146
Negative	10.4 (5.7)	9.9 (3.8)	0.862
General	18.4 (8.5)	21.5 (4.8)	**<0.001**
Depressive	6.0 (3.9)	6.8 (2.7)	**0.040**
Total	38.7 (15.5)	40.3 (9.3)	0.139
**Antipsychotic treatment, mean dose (SD)**[2]			
Chlorpromazine equivalent dose (mg) [#]	322.2 (172.2)	977.6 (1869.1)	**<0.001**
Chlorpromazine equivalent dose, imputed (mg)	349.2 (303.2)	949.2 (1693.6)	**<0.001**
Clozapine, yes (%)[1]	12 (5.8)	33 (31.7)	**<0.001**
**Depression symptoms, mean (SD)**[#]			
Hamilton Depression Rating Scale, mean (SD)	-	4.8 (4.8)	-
Calgary Depression Scale for schizophrenia, mean (SD)	5.4 (4.6)	-	-
**General cognitive performance, mean (SD)**[2+#]			
Verbal	94.1 (16.4)	97.9 (18.9)	0.130
Performance	85.9 (14.0)	87.9 (16.5)	0.286
General cognitive performance as sum of verbal and performance IQ scores (Full Scale IQ scores)	90.0 (14.9)	93.2 (16.9)	0.120
**Cytokine (pg/mL)**			
Plasma IL-6 (raw), **median (IQR), unadjusted model**[4#]	0.7 (0.4 – 1.0)	3.4 (1.4 – 3.7)	**<0.001**

Plasma IL-6 (raw), **median (IQR), adjusted model**[5#]	0.7 (0.4 – 1.0)	3.4 (1.4 – 3.7)	**<0.001**

* Data for one individual with Early schizophrenia (BeneMin study) was not available (total n=206).

[1] Pearson's Chi-square test; [2] Mann-Whitney U Test; [3] Fisher's Exact Test (used for comparisons of categorical data when expected frequencies in contingency tables were less than 5). [4]Generalized Linear Model (GLM). IL-6 levels were natural log and z-scored, while the raw descriptive statistics is provided. [5] GLM adjusted for age, sex, and chlorpromazine equivalent dose (imputed measure). IL-6 levels were natural log and z-scored, while the raw descriptive statistics is provided.

[#] Missing data: age (Early schizophrenia, n=1); years of education (Early schizophrenia, n=71; Established schizophrenia, n=8); body mass index (Early schizophrenia, n=3; Established schizophrenia, n=3); diagnosis (Early schizophrenia, n=4; Established schizophrenia, n=1); illness onset age (Established schizophrenia, n=5); illness duration (Established schizophrenia, n=5); PANSS positive (Established schizophrenia, n=3); PANSS negative (Established schizophrenia, n=3); PANSS general (Early schizophrenia, n=1; Established schizophrenia, n=3); PANSS total (Early schizophrenia, n=1; Established schizophrenia, n=3); PANSS depression (Established schizophrenia, n=2); Chlorpromazine equivalent dose (Early schizophrenia, n=59; Established schizophrenia, n=20); Hamilton Depression Rating Scale (Established schizophrenia, n=1); Calgary Depression Scale for schizophrenia (Established schizophrenia, n=1); Verbal (Early schizophrenia, n=6); Performance (Early schizophrenia, n=29); Full Scale IQ scores (Early schizophrenia, n=5); plasma IL-6 (Early schizophrenia, n=5; Established schizophrenia, n=8).

[+] Wechsler Adult Intelligence Scale-Third Edition (WAIS-III-R) shortened and prorated version of WAIS-III.

Significant results are depicted in bold (p<0.05).

Abbreviations: IL-6, Interleukin 6; IQ: Intelligence Quotient; PANSS: Positive and Negative Syndrome Scale; SD: Standard Deviation; IQR: Interquartile Range

Table S5A. Composite score loadings in the Confirmatory Factor Analysis with plasma IL-6 levels, structural brain metrics, and general cognitive performance measure, and in individuals with Early (BeneMin sample; n=102) and Established schizophrenia (iRELATE sample; n=42) combined

**Composite scores**	**Indicator**	**B**	**SE**	**Z**	**P**	**Beta**
plasma IL-6	plasma IL-6	1	0.059	16.971	**<0.001**	1
Structural brain	Cortical thickness and volume	0.973	0.057	16.971	**<0.001**	1
General cognitive performance	Verbal and performance estimates	0.808	0.048	16.971	**<0.001**	1

Abbreviations: B: unstandardised estimates of path coefficient composite scores; Beta, standardised estimates of path coefficient composite scores; SE, standard error.

Significant results are depicted in bold (p<0.05).

Table S5B. Composite score loadings in the Confirmatory Factor Analysis with plasma IL-6 levels, structural brain metrics, and general cognitive performance measure, in Early schizophrenia only (BeneMin sample; n=102)

**Composite scores**	**Indicator**	**B**	**SE**	**Z**	**P**	**Beta**
plasma IL-6	plasma IL-6	1	0.070	14.283	**<0.001**	1
Structural brain	Cortical thickness and volume	0.978	0.069	14.283	**<0.001**	1
General cognitive performance	Verbal and performance estimates	0.801	0.056	14.283	**<0.001**	1

Abbreviations: B: unstandardised estimates of path coefficient composite scores; Beta, standardised estimates of path coefficient composite scores; SE, standard error.

Significant results are depicted in bold (p<0.05).

Table S6A. Model PANSS depression in individuals with Early schizophrenia (BeneMin; n=102) and Established schizophrenia (iRELATE; n=42) combined

**Confirmatory factor analysis – standardised estimates for relations among the composite scores**						
		**Statistics**				
**Composite score I**	**Composite score II**	**B**	**SE**	**Z**	**P**	**Beta**
Plasma IL-6	Structural brain	**-0.247**	**0.078**	**-3.163**	**0.002**	**-0.247**
Plasma IL-6	General cognitive performance	-0.027	0.083	-0.327	0.744	-0.027
Structural brain	General cognitive performance	**0.353**	**0.073**	**4.842**	**<0.001**	**0.353**
**CFA fit indices**		$X^2 = 0$; CFI = 1; TLI = 1; RMSEA = 0; SRMR = 0				
**Outcome: PANSS depression symptom severity**		**Statistics (unadjusted model)**				
**Composite score I**	**Composite score II**	**B**	**SE**	**Z**	**P**	**Beta**
Plasma IL-6	PANSS Depression	0.091	0.085	1.064	0.287	0.091
Structural brain	PANSS Depression	-0.022	0.085	-0.262	0.793	-0.022
**SEM fit indices**		$X^2 = 0.692$; CFI = 1; TLI = 1.074; RMSEA = 0; SRMR = 0.017				
**Confirmatory factor analysis – standardised estimates for relations among the composite scores**						
		**Statistics**				
**Composite score I**	**Composite score II**	**B**	**SE**	**Z**	**P**	**Beta**
Plasma IL-6	Structural brain	**-0.247**	**0.078**	**-3.163**	**0.002**	-0.247
Plasma IL-6	General cognitive performance	-0.027	0.083	-0.327	0.744	-0.027
Structural brain	General cognitive performance	**0.353**	**0.073**	**4.842**	**<0.001**	**0.353**
**CFA fit indices**		$X^2 = 0$; CFI = 1; TLI = 1; RMSEA = 0; SRMR = 0				
**Outcome: PANSS depression symptom severity**		**Statistics (adjusted model)***				
**Composite score I**	**Composite score II**	**B**	**SE**	**Z**	**P**	**Beta**
Plasma IL-6	PANSS depression	0.065	0.096	0.681	0.496	0.065
Structural brain	PANSS depression	0.001	0.094	0.01	0.992	0.001
**SEM fit indices**		$X^2 = 5.972$; CFI = 1; TLI = 1.104; RMSEA = 0; SRMR = 0.03				

Abbreviations: B: unstandardised estimates of path coefficient composite scores; Beta, standardised estimates of path coefficient composite scores; SE, standard error.

* Adjusted for age, sex, and chlorpromazine equivalent dose (imputed measure).

Significant results are depicted in bold (p<0.05).

Table S6B. Model PANSS depression in individuals with Early schizophrenia only (BeneMin sample; n=102)

**Confirmatory factor analysis – standardised estimates for relations among the composite scores**						
		**Statistics**				
**Composite score I**	**Composite score II**	**B**	**SE**	**Z**	**P**	**Beta**
Plasma IL-6	Structural brain	**-0.274**	**0.092**	**-2.992**	**0.003**	**-0.274**
Plasma IL-6	General cognitive performance	-0.013	0.099	-0.001	0.999	-0.013
Structural brain	General cognitive performance	**0.303**	**0.090**	**3.363**	**0.001**	**0.303**
**CFA fit indices**		$X^2 = 0$; CFI = 1; TLI = 1; RMSEA = 0; SRMR = 0				
**Outcome: PANSS depression symptom severity**		**Statistics (unadjusted model)**				
**Composite score I**	**Composite score II**	**B**	**SE**	**Z**	**P**	**Beta**
Plasma IL-6	PANSS depression	0.130	0.102	1.272	0.203	0.130
Structural brain	PANSS depression	-0.031	0.101	-0.309	0.757	-0.032
**SEM fit indices**		$X^2 = 0.783$; CFI = 1; TLI = 1.084; RMSEA = 0; SRMR = 0.022				
**Confirmatory factor analysis – standardised estimates for relations among the composite scores**						
		**Statistics**				
**Composite score I**	**Composite score II**	**B**	**SE**	**Z**	**P**	**Beta**
Plasma IL-6	Structural brain	**-0.274**	**0.092**	**-2.992**	**0.003**	**-0.274**
Plasma IL-6	General cognitive performance	-0.013	0.099	-0.001	0.999	-0.013
Structural brain	General cognitive performance	**0.303**	**0.090**	**3.363**	**0.001**	**0.303**
**CFA fit indices**		$X^2 = 0$; CFI = 1; TLI = 1; RMSEA = 0; SRMR = 0				
**Outcome: PANSS depression symptom severity**		**Statistics (adjusted model)***				
**Composite score I**	**Composite score II**	**B**	**SE**	**Z**	**P**	**Beta**
Plasma IL-6	PANSS depression	0.139	0.104	1.335	0.182	0.139
Structural brain	PANSS depression	-0.041	0.101	-0.409	0.682	-0.042
**SEM fit indices**		$X^2 = 12.867$; CFI = 0.830; TLI = 0.694; RMSEA = 0.053; SRMR = 0.055				

Abbreviations: B: unstandardised estimates of path coefficient composite scores; Beta, standardised estimates of path coefficient composite scores; SE, standard error.

* Adjusted for age, sex, and chlorpromazine equivalent dose (imputed measure).

Significant results are depicted in bold (p<0.05).

Table S7A. Model PANSS negative in individuals with Early schizophrenia (BeneMin; n=102) and Established schizophrenia (iRELATE; n=42) combined

**Confirmatory factor analysis – standardised estimates for relations among the composite scores**						
		**Statistics**				
**Composite score I**	**Composite score II**	**B**	**SE**	**Z**	**P**	**Beta**
Plasma IL-6	Structural brain	**-0.247**	**0.078**	**-3.163**	**0.002**	**-0.247**
Plasma IL-6	General cognitive performance	-0.027	0.083	-0.327	0.744	-0.027
Structural brain	General cognitive performance	**0.353**	**0.073**	**4.842**	**<0.001**	**0.353**
**CFA fit indices**		$X^2 = 0$; CFI = 1; TLI = 1; RMSEA = 0; SRMR = 0				
**Outcome: PANSS Negative symptom severity**		**Statistics (unadjusted model)**				
**Composite score I**	**Composite score II**	**B**	**SE**	**Z**	**P**	**Beta**
Plasma IL-6	PANSS negative	0.075	0.085	0.879	0.380	0.075
Structural brain	PANSS negative	-0.046	0.085	-0.534	0.593	-0.046
**SEM fit indices**		$X^2 = 5.067$; CFI = 0.861; TLI = 0.169; RMSEA = 0.168; SRMR = 0.046				
**Confirmatory factor analysis – standardised estimates for relations among the composite scores**						
		**Statistics**				
**Composite score I**	**Composite score II**	**B**	**SE**	**Z**	**P**	**Beta**
Plasma IL-6	Structural brain	**-0.247**	**0.078**	**-3.163**	**0.002**	**-0.247**
Plasma IL-6	General cognitive performance	-0.027	0.083	-0.327	0.744	-0.027
Structural brain	General cognitive performance	**0.353**	**0.073**	**4.842**	**<0.001**	**0.353**
**CFA fit indices**		$X^2 = 0$; CFI = 1; TLI = 1; RMSEA = 0; SRMR = 0				
**Outcome: PANSS Negative symptom severity (adjusted)***		**Statistics (adjusted model)***				
**Composite score I**	**Composite score II**	**B**	**SE**	**Z**	**P**	**Beta**
Plasma IL-6	PANSS negative	0.120	0.097	1.236	0.216	0.120
Structural brain	PANSS negative	-0.086	0.096	-0.898	0.369	-0.086
**SEM fit indices**		$X^2 = 12.463$; CFI = 0.954; TLI = 0.892; RMSEA = 0.052; SRMR = 0.044				

Abbreviations: B: unstandardised estimates of path coefficient composite scores; Beta, standardised estimates of path coefficient composite scores; SE, standard error.

* Adjusted for age, sex, and chlorpromazine equivalent dose (imputed measure).

Significant results are depicted in bold (p<0.05).

Table S7B. Model PANSS negative in individuals with Early schizophrenia only (BeneMin sample; n=102)

**Confirmatory factor analysis – standardised estimates for relations among the composite scores**						
		**Statistics**				
**Composite score I**	**Composite score II**	**B**	**SE**	**Z**	**P**	**Beta**
Plasma IL-6	Structural brain	**-0.274**	**0.092**	**-2.992**	**0.003**	**-0.274**
Plasma IL-6	General cognitive performance	-0.013	0.099	-0.013	0.999	-0.013
Structural brain	General cognitive performance	**0.303**	**0.090**	**3.363**	**0.001**	**0.303**
**CFA fit indices**		$X^2 = 0$; CFI = 1; TLI = 1; RMSEA = 0; SRMR = 0				
**Outcome: PANSS negative symptom severity**		**Statistics (unadjusted model)**				
**Composite score I**	**Composite score II**	**B**	**SE**	**Z**	**P**	**Beta**
Plasma IL-6	PANSS negative	0.078	0.102	0.764	0.445	0.078
Structural brain	PANSS negative	-0.043	0.102	-0.423	0.672	-0.043
**SEM fit indices**		$X^2 = 2.424$; CFI = 0.911; TLI = 0.467; RMSEA = 0.118; SRMR = 0.039				
**Confirmatory factor analysis – standardised estimates for relations among the composite scores**						
		**Statistics**				
**Composite score I**	**Composite score II**	**B**	**SE**	**Z**	**P**	**Beta**
Plasma IL-6	Structural brain	**-0.274**	**0.092**	**-2.992**	**0.003**	**-0.274**
Plasma IL-6	General cognitive performance	-0.013	0.099	-0.013	0.999	-0.013
Structural brain	General cognitive performance	**0.303**	**0.090**	**3.363**	**0.001**	**0.303**
**CFA fit indices**		$X^2 = 0$; CFI = 1; TLI = 1; RMSEA = 0; SRMR = 0				
**Outcome: PANSS negative symptom severity(adjusted)***		**Statistics (adjusted model)***				
**Composite score I**	**Composite score II**	**B**	**SE**	**Z**	**P**	**Beta**
Plasma IL-6	PANSS negative	0.115	0.103	1.117	0.264	0.115
Structural brain	PANSS negative	-0.037	0.100	-0.370	0.711	-0.037
**SEM fit indices**		$X^2 = 16.127$; CFI = 0.726; TLI = 0.508; RMSEA = 0.078; SRMR = 0.059				

Abbreviations: B: unstandardised estimates of path coefficient composite scores; Beta, standardised estimates of path coefficient composite scores; SE, standard error.

* Adjusted for age, sex, and chlorpromazine equivalent dose (imputed measure).

Significant results are depicted in bold (p<0.05).

Figure S1A

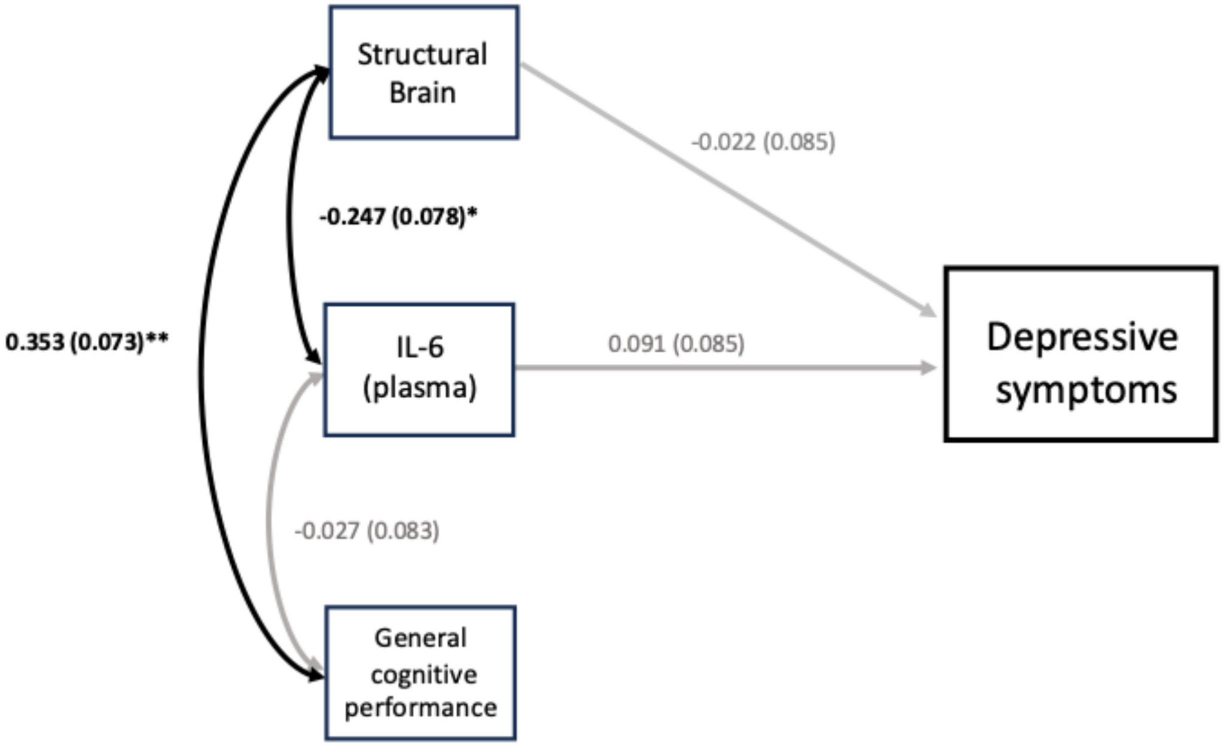

Figure S1A. Structural Equation Modeling – Early (BeneMin) and Established (iRELATE) samples combined.

Model PANSS depression (unadjusted).

Individuals with Early schizophrenia (n=102) and Established schizophrenia combined (n=42). Estimates are standardized path coefficient composite scores. Single-headed arrows represent composite regression paths. Double-headed arrows depict covariances between the three endogenous variables. Black bold arrows denote significant associations (*p < 0.05; **p < 0.001). Grey arrows represent non-significant associations.

Factor Score Regression was used to generate composite scores for analyses.

CFA fit indices: $X^2 = 0$; CFI = 1; TLI = 1; RMSEA = 0; SRMR = 0.

SEM fit indices: $X^2 = 0.692$; CFI = 1; TLI = 1.074; RMSEA = 0; SRMR = 0.017.

Abbreviations: plasma IL-6: Interleukin-6; CFI: Comparative Fix Index; TLI: Tucker-Lewis Index; RMSEA: Root Mean Squared Error of Approximation; SRMR: Standardized Root Mean Square Residual.

Figure S1B

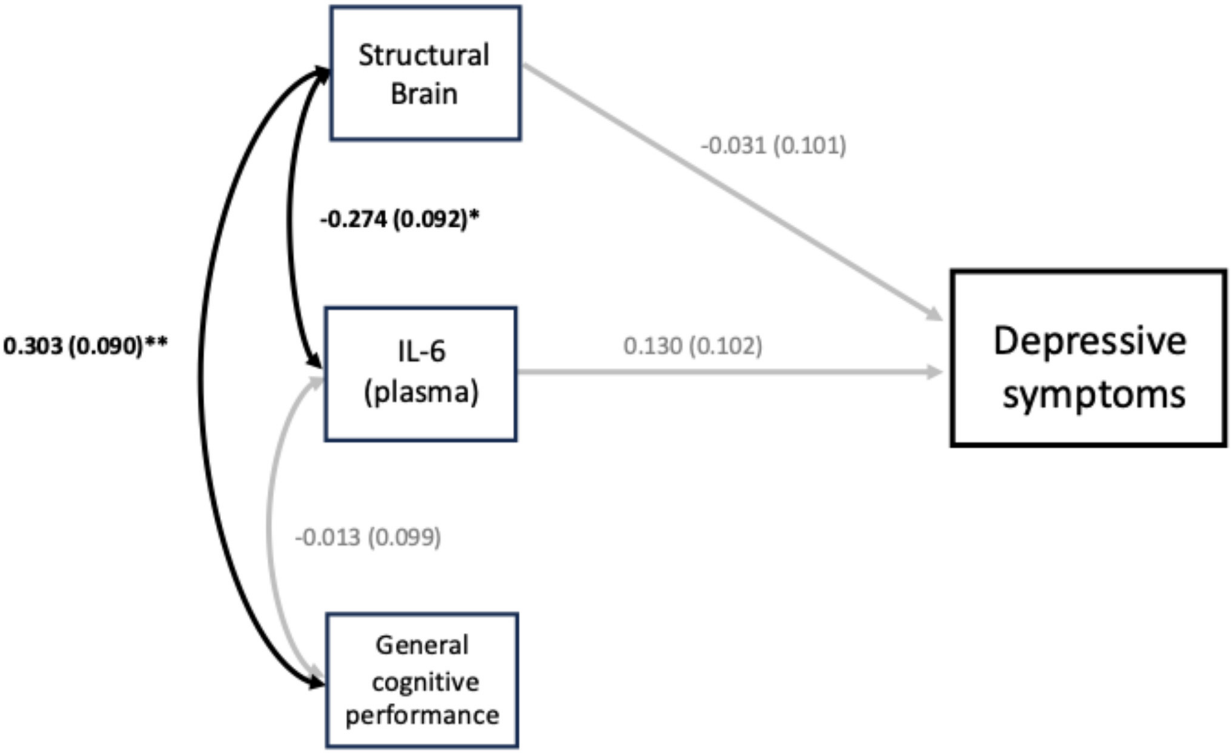

Figure S1B. Structural Equation Modeling – Early schizophrenia only (BeneMin sample).
Model PANSS depression (unadjusted).
Individuals with Early schizophrenia only (n=102).
Estimates are standardized path coefficient composite scores. Single-headed arrows represent composite regression paths. Double-headed arrows depict covariances between the three endogenous variables. Black bold arrows denote significant associations (*p < 0.05; **p < 0.001). Grey arrows represent non-significant associations.
Factor Score Regression was used to generate composite scores for analyses.
CFA fit indices: $X^2 = 0$; CFI = 1; TLI = 1; RMSEA = 0; SRMR = 0.
SEM fit indices: $X^2 = 0.783$; CFI = 1; TLI = 1.084; RMSEA = 0; SRMR = 0.022.
Abbreviations: plasma IL-6: Interleukin-6; CFI: Comparative Fix Index; TLI: Tucker-Lewis Index; RMSEA: Root Mean Squared Error of Approximation; SRMR: Standardized Root Mean Square Residual.

Figure S2A

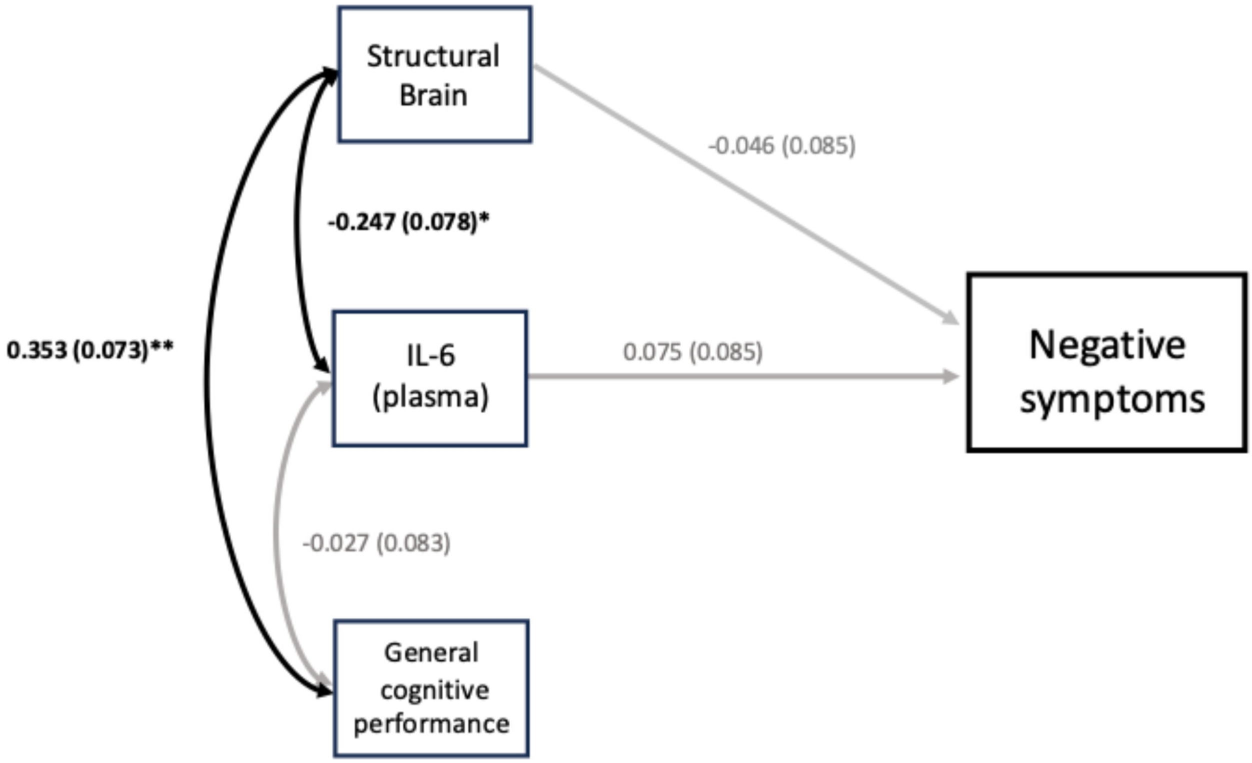

Figure S2A. Structural Equation Modeling – Early (BeneMin) and Established (iRELATE) samples combined.

Model PANSS negative (unadjusted).

Individuals with Early schizophrenia (n=102) and Established schizophrenia combined (n=42). Estimates are standardized path coefficient composite scores. Single-headed arrows represent composite regression paths. Double-headed arrows depict covariances between the three endogenous variables. Black bold arrows denote significant associations (*p < 0.05; **p < 0.001). Grey arrows represent non-significant associations.

Factor Score Regression was used to generate composite scores for analyses.

CFA fit indices: $X^2 = 0$; CFI = 1; TLI = 1; RMSEA = 0; SRMR = 0.

SEM fit indices: $X^2 = 5.067$; CFI = 0.861; TLI = 0.169; RMSEA = 0.168; SRMR = 0.046.

Abbreviations: plasma IL-6: Interleukin-6; CFI: Comparative Fix Index; TLI: Tucker-Lewis Index; RMSEA: Root Mean Squared Error of Approximation; SRMR: Standardized Root Mean Square Residual.

Figure S2B

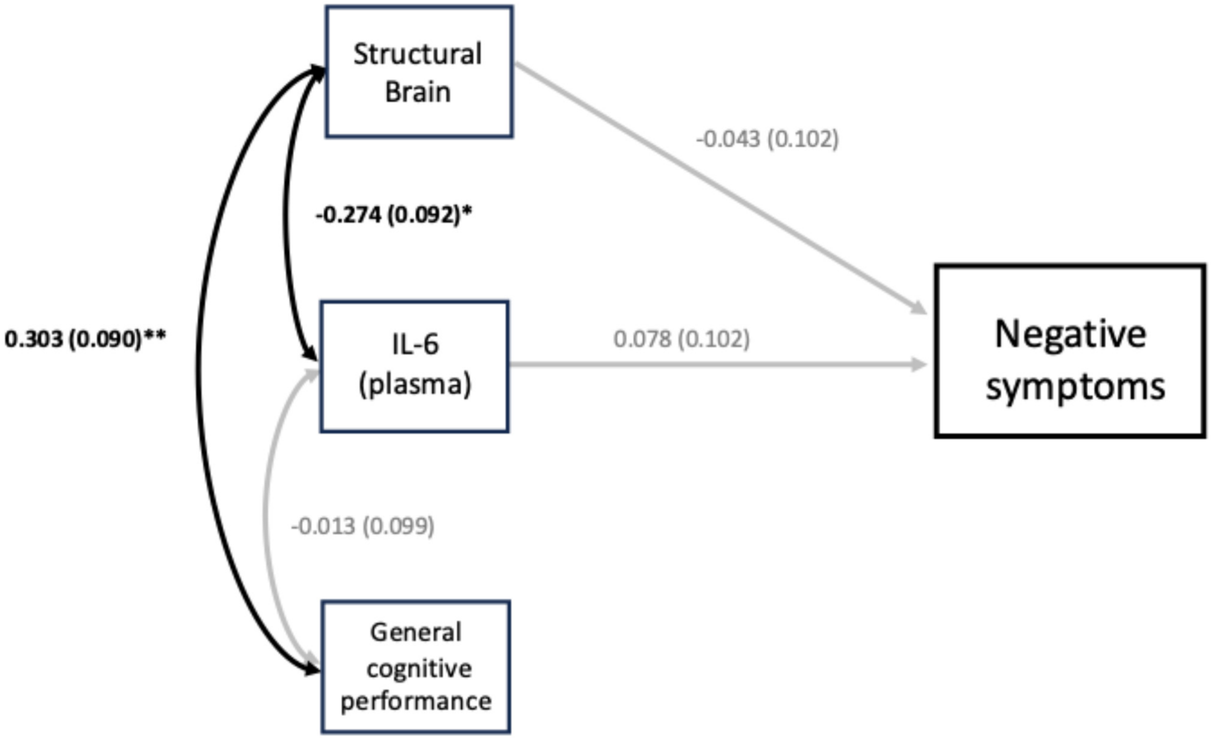

Figure S2B. Structural Equation Modeling – Early schizophrenia (BeneMin) sample only.
Model PANSS negative (unadjusted).
Individuals with Early schizophrenia only (n=102).
Estimates are standardized path coefficient composite scores. Single-headed arrows represent composite regression paths. Double-headed arrows depict covariances between the three endogenous variables. Black bold arrows denote significant associations (*p < 0.05; **p < 0.001). Grey arrows represent non-significant associations.
Factor Score Regression was used to generate composite scores for analyses.
CFA fit indices: $X^2 = 0$; CFI = 1; TLI = 1; RMSEA = 0.118; SRMR = 0.039.
SEM fit indices: $X^2 = 2.424$; CFI = 0.911; TLI = 0.467; RMSEA = 0.168; SRMR = 0.046.
Abbreviations: plasma IL-6: Interleukin-6; CFI: Comparative Fix Index; TLI: Tucker-Lewis Index; RMSEA: Root Mean Squared Error of Approximation; SRMR: Standardized Root Mean Square Residual.

References

1. van Vliet IM, de Beurs E (2007): [The MINI-International Neuropsychiatric Interview. A brief structured diagnostic psychiatric interview for DSM-IV en ICD-10 psychiatric disorders]. *Tijdschr Psychiatr* 49: 393–7.
2. Kay SR, Fiszbein A, Opler LA (1987): The positive and negative syndrome scale (PANSS) for schizophrenia. *Schizophr Bull*. https://doi.org/10.1093/schbul/13.2.261
3. Dauvermann MR, Mothersill D, Rokita KI, King S, Holleran L, Kane R, *et al.* (2021): Changes in Default-Mode Network Associated With Childhood Trauma in Schizophrenia. *Schizophr Bull* 47: 1482–1494.
4. King S, Holleran L, Mothersill D, Patlola S, Rokita K, McManus R, *et al.* (2021): Early life Adversity, functional connectivity and cognitive performance in Schizophrenia: The mediating role of IL-6. *Brain Behav Immun* 98. https://doi.org/10.1016/j.bbi.2021.06.016
5. Rokita KI, Holleran L, Dauvermann MR, Mothersill D, Holland J, Costello L, *et al.* (2020): Childhood trauma, brain structure and emotion recognition in patients with schizophrenia and healthy participants. *Soc Cogn Affect Neurosci*. https://doi.org/10.1093/SCAN/NSAA160
6. First MB, Spitzer RL, Gibbon Miriam, Williams JBW (1997): *Structured Clinical Interview for DSM-IV Axis I Disorders SCID-I : Clinician Version, Administration Booklet*. American Psychiatric Publishing.
7. Kay SR, Fiszbein A, Opler LA (1987): The positive and negative syndrome scale (PANSS) for schizophrenia. *Schizophr Bull* 13: 261–76.
8. Wymer JH, Rayls K, Wagner MT (2003): Utility of a clinically derived abbreviated form of the WAIS-III. *Archives of Clinical Neuropsychology*. https://doi.org/10.1016/S0887-6177(02)00221-4
9. Blyler CR, Gold JM, Iannone VN, Buchanan RW (2000): Short form of the WAIS-III for use with patients with schizophrenia. *Schizophr Res* 46: 209–215.
10. Watson AJ, Giordano A, Suckling J, Barnes TRE, Husain N, Jones PB, *et al.* (2023): Cognitive function in early-phase schizophrenia-spectrum disorder: IQ subtypes, brain volume and immune markers. *Psychol Med* 53: 2842.
11. Dauvermann MR, Donohoe G (2018): The role of childhood trauma in cognitive performance in schizophrenia and bipolar disorder - A systematic review. *Schizophr Res Cogn* 16: 1–11.
12. Deakin B, Suckling J, Barnes TRE, Byrne K, Chaudhry IB, Dazzan P, *et al.* (2018): The benefit of minocycline on negative symptoms of schizophrenia in patients with recent-onset psychosis (BeneMin): a randomised, double-blind, placebo-controlled trial. *Lancet Psychiatry* 5: 885–894.
13. Lisiecka DM, Suckling J, Barnes TRE, Chaudhry IB, Dazzan P, Husain N, *et al.* (2015): The benefit of minocycline on negative symptoms in early-phase psychosis in addition to standard care - extent and mechanism (BeneMin): Study protocol for a randomised controlled trial. *Trials* 16: 1–15.
14. Suckling J, Barnes A, Job D, Brennan D, Lymer K, Dazzan P, *et al.* (2012): The neuro/PsyGRID calibration experiment: Identifying sources of variance and bias in multicenter MRI studies. *Hum Brain Mapp* 33: 373–386.
15. Seiger R, Ganger S, Kranz GS, Hahn A, Lanzenberger R (2018): Cortical Thickness Estimations of FreeSurfer and the CAT12 Toolbox in Patients with Alzheimer's Disease and Healthy Controls. *J Neuroimaging* 28: 515–523.

16. Ay U, Kizilates-Evin G, Bayram A, Kurt E, Demiralp T (2022): Comparison of FreeSurfer and CAT12 Software in Parcel-Based Cortical Thickness Calculations. *Brain Topogr* 35: 572–582.
17. Gaser C, Dahnke R, Thompson PM, Kurth F, Luders E, Initiative ADN (2023): CAT – A Computational Anatomy Toolbox for the Analysis of Structural MRI Data. *bioRxiv* 2022.06.11.495736.
18. Desikan RS, Ségonne F, Fischl B, Quinn BT, Dickerson BC, Blacker D, *et al.* (2006): An automated labeling system for subdividing the human cerebral cortex on MRI scans into gyral based regions of interest. *Neuroimage* 31: 968–980.
19. DiStefano C, Zhu M, Mîndrilă D (2009): Understanding and using factor scores: Considerations for the applied researcher. *Practical Assessment, Research and Evaluation*.
20. Lu IRR, Kwan E, Thomas DR, Cedzynski M (2011): Two new methods for estimating structural equation models: An illustration and a comparison with two established methods. *International Journal of Research in Marketing*. https://doi.org/10.1016/j.ijresmar.2011.03.006
21. Kline, Rex B (2011): Principles and Practice of Structural Equation Modeling, 3rd edition Guilford Press. *The Guilford Press New York London*.
22. Nash JC (2014): On Best Practice Optimization Methods in R. *J Stat Softw* 60: 1–14.
23. Austin PC, White IR, Lee DS, van Buuren S (2020): Missing Data in Clinical Research: A Tutorial on Multiple Imputation. *Canadian Journal of Cardiology*. https://doi.org/10.1016/j.cjca.2020.11.010
24. Kose M, Pariante CM, Dazzan P, Mondelli V (2021): The Role of Peripheral Inflammation in Clinical Outcome and Brain Imaging Abnormalities in Psychosis: A Systematic Review. *Frontiers in Psychiatry*. https://doi.org/10.3389/fpsyt.2021.612471
25. Alexandros Lalousis P, Schmaal L, Wood SJ, L.E.P Reniers R, Cropley VL, Watson A, *et al.* (2023): Inflammatory subgroups of schizophrenia and their association with brain structure: A semi-supervised machine learning examination of heterogeneity. *Brain Behav Immun* 113: 166–175.
26. Wu D, Lv P, Li F, Zhang W, Fu G, Dai J, *et al.* (2019): Association of peripheral cytokine levels with cerebral structural abnormalities in schizophrenia. *Brain Res* 1724. https://doi.org/10.1016/J.BRAINRES.2019.146463
27. Williams JA, Burgess S, Suckling J, Lalousis PA, Batool F, Griffiths SL, *et al.* (2022): Inflammation and Brain Structure in Schizophrenia and Other Neuropsychiatric Disorders: A Mendelian Randomization Study. *JAMA Psychiatry* 79. https://doi.org/10.1001/jamapsychiatry.2022.0407
28. Morrens M, Overloop C, Coppens V, Loots E, Van Den Noortgate M, Vandenameele S, *et al.* (2022): The relationship between immune and cognitive dysfunction in mood and psychotic disorder: a systematic review and a meta-analysis. *Molecular Psychiatry 2022* 1–10.
29. Patlola SR, Donohoe G, McKernan DP (2023): The relationship between inflammatory biomarkers and cognitive dysfunction in patients with schizophrenia: A systematic review and meta-analysis. *Prog Neuropsychopharmacol Biol Psychiatry* 121: 110668.
30. Chang M, Womer FY, Edmiston EK, Bai C, Zhou Q, Jiang X, *et al.* (2018): Neurobiological Commonalities and Distinctions among Three Major Psychiatric Diagnostic Categories: A Structural MRI Study. *Schizophr Bull*. https://doi.org/10.1093/schbul/sbx028
31. Hettwer MD, Larivière S, Park BY, van den Heuvel OA, Schmaal L, Andreassen OA, *et al.* (2022): Coordinated cortical thickness alterations across six neurodevelopmental and psychiatric disorders. *Nat Commun* 13: 6851.

32. Patel Y, Parker N, Shin J, Howard D, French L, Thomopoulos SI, *et al.* (2020): Virtual Histology of Cortical Thickness and Shared Neurobiology in 6 Psychiatric Disorders. *JAMA Psychiatry*. https://doi.org/10.1001/jamapsychiatry.2020.2694
33. Yang Y, Li X, Cui Y, Liu K, Qu H, Lu Y, *et al.* (2022): Reduced Gray Matter Volume in Orbitofrontal Cortex Across Schizophrenia, Major Depressive Disorder, and Bipolar Disorder: A Comparative Imaging Study. *Front Neurosci* 16: 750.
34. Goodkind M, Eickhoff SB, Oathes DJ, Jiang Y, Chang A, Jones-Hagata LB, *et al.* (2015): Identification of a common neurobiological substrate for mental Illness. *JAMA Psychiatry*. https://doi.org/10.1001/jamapsychiatry.2014.2206
35. Bekhbat M, Treadway MT, Felger JC (2022): Inflammation as a Pathophysiologic Pathway to Anhedonia: Mechanisms and Therapeutic Implications. *Current Topics in Behavioral Neurosciences. Springer, Berlin, Heidelberg.* https://doi.org/10.1007/7854_2021_294
